# Regulatory factor X1 induces macrophage M1 polarization by promoting DNA demethylation in autoimmune inflammation

**DOI:** 10.1172/jci.insight.165546

**Published:** 2023-10-23

**Authors:** Shuang Yang, Pei Du, Haobo Cui, Meiling Zheng, Wei He, Xiaofei Gao, Zhi Hu, Sujie Jia, Qianjin Lu, Ming Zhao

**Affiliations:** 1 Department of Dermatology, Second Xiangya Hospital, Central South University, Hunan Key Laboratory of Medical Epigenomics, Changsha, China.; 2 Research Unit of Key Technologies of Diagnosis and Treatment for Immune-related Skin Diseases, Chinese Academy of Medical Sciences, Central South University, Changsha, China.; 3 Department of Pharmacy, The Third Xiangya Hospital, Central South University, Changsha, China.; 4 Institute of Dermatology, Chinese Academy of Medical Sciences and Peking Union Medical College, Nanjing, China.; 5 Key Laboratory of Basic and Translational Research on Immune-Mediated Skin Diseases, Chinese Academy of Medical Sciences, Nanjing, China.

**Keywords:** Autoimmunity, Inflammation, Macrophages, Transcription

## Abstract

Abnormal macrophage polarization is generally present in autoimmune diseases. Overwhelming M1 macrophage activation promotes the continuous progression of inflammation, which is one of the reasons for the development of autoimmune diseases. However, the underlying mechanism is still unclear. Here we explore the function of Regulatory factor X1 (RFX1) in macrophage polarization by constructing colitis and lupus-like mouse models. Both in vivo and in vitro experiments confirmed that RFX1 can promote M1 and inhibit M2 macrophage polarization. Furthermore, we found that RFX1 promoted DNA demethylation of macrophage polarization–related genes by increasing APOBEC3A/Apobec3 expression. We identified a potential RFX1 inhibitor, adenosine diphosphate (ADP), providing a potential strategy for treating autoimmune diseases.

## Graphical abstract

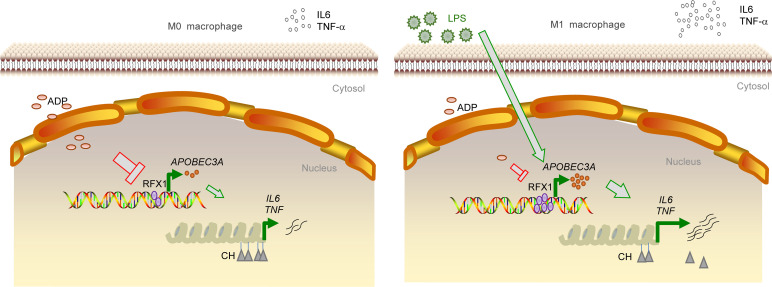

## Introduction

Various autoimmune diseases are characterized by unsolved inflammation. Systemic lupus erythematosus (SLE) is a kind of systemic autoimmune disease with inflammation in multiple organs, mainly including skin, kidneys, joints, and the nervous system (1). Inflammatory bowel diseases (IBDs), including Crohn’s disease and ulcerative colitis, belong to organ-specific autoimmune disorders characterized by unresolved inflammation in the gastrointestinal tract (2). Macrophages play a vital role in the whole inflammatory process, including initiation, inflammation, resolution, and tissue restoration (3). Macrophages were distributed in almost all organs and participated in the occurrence and development of autoimmune diseases by phagocytosis, antigen presentation, and cytokine secretion (4, 5).

Under environmental signals, macrophages exhibit a high degree of plasticity and heterogeneity and generally tend to differentiate into 2 main phenotypes — classically activated macrophage (M1) and alternately activated macrophage (M2), termed macrophage polarization. The M1 macrophage shows an inflammatory phenotype mainly induced by LPS or IFN-γ, characterized by highly expressing proinflammatory factors such as IL-6, IL-1β, and TNF-α, and cell-surface molecules such as CD86 and MHCII, contributing to inflammation and tissue damage (6, 7). The M2 macrophage highly expressing CD163, CD206, Arg-1, and other markers shows an antiinflammatory phenotype induced by Th2 cytokines such as IL-4, contributing to inflammation elimination and tissue repair (8). Proinflammatory M1 macrophage facilitated by circulating microparticles from patients with autoimmune diseases such as SLE can promote T cell activation and induce B cell activation and survival (9). M1 macrophages could enter the injured kidney to promote apoptotic death of tubular cells, which aggravated the kidney damage (10). Adoptive transplantation of M1 macrophages aggravated clodronate-induced augmentation in SLE severity, while M2 macrophage alleviated it (11). In addition, the M1/M2 ratio also increased in the lamina propria of the inflamed intestine in patients with IBD, promoting activation of the local macrophages to favor intestinal inflammation and destroy epithelial barrier integrity (12, 13). Transference of M2 macrophage ameliorated the chronic inflammation in animal models of IBD (14, 15).

Various studies have found some transcription factors to participate in regulating macrophage polarization. For example, the Kruppel-like factor 5 (KLF5) directly activated the transcription of a variety of proinflammatory genes to promote M1 macrophage polarization and played an important role in the pathogenesis of LPS-induced endotoxin shock mice (16). Inhibition of SOCS3 expression could induce M2 macrophage polarization to inhibit inflammation and promote the functional recovery of the rat with intracerebral hemorrhage (17). However, the mechanism of abnormal proinflammatory macrophage activation in autoimmune diseases remains unclear. Regulatory factor X1 (RFX1) can bind to the *cis*-acting element X-box, which contains a C-terminal inhibitory domain and an N-terminal activation domain, to obtain the dual ability to inhibit and activate the transcription of the target gene (18, 19). Our previous study found that the downregulation of RFX1 in CD4^+^ T cells from peripheral blood of patients with SLE promoted T cell autoreactive activation and increased Th17 cell differentiation, which is involved in the pathogenesis of SLE (20–22). The roles of RFX1 in macrophages and autoimmune disease have not been reported.

In this study, we found that the RFX1 expression was increased in M1 macrophages and correlated with M1-related gene expression. The in vitro and in vivo experiments showed that RFX1 increased M1 macrophage polarization and promoted the pathogenesis of colitis or lupus in mice. Mechanistically, through increasing APOBEC3A expression, RFX1 facilitated autoimmune inflammation by promoting DNA demethylation of M1-related genes *IL6* and *TNF*. We further identified that adenosine diphosphate (ADP), as an effective inhibitor, inhibits the function of RFX1 in macrophage polarization. Collectively, our results reveal the role of RFX1 in macrophage polarization and provide a potential target for autoimmune inflammation.

## Results

### RFX1 expression was increased in inflammatory and autoimmune diseases.

**Figure 1:**
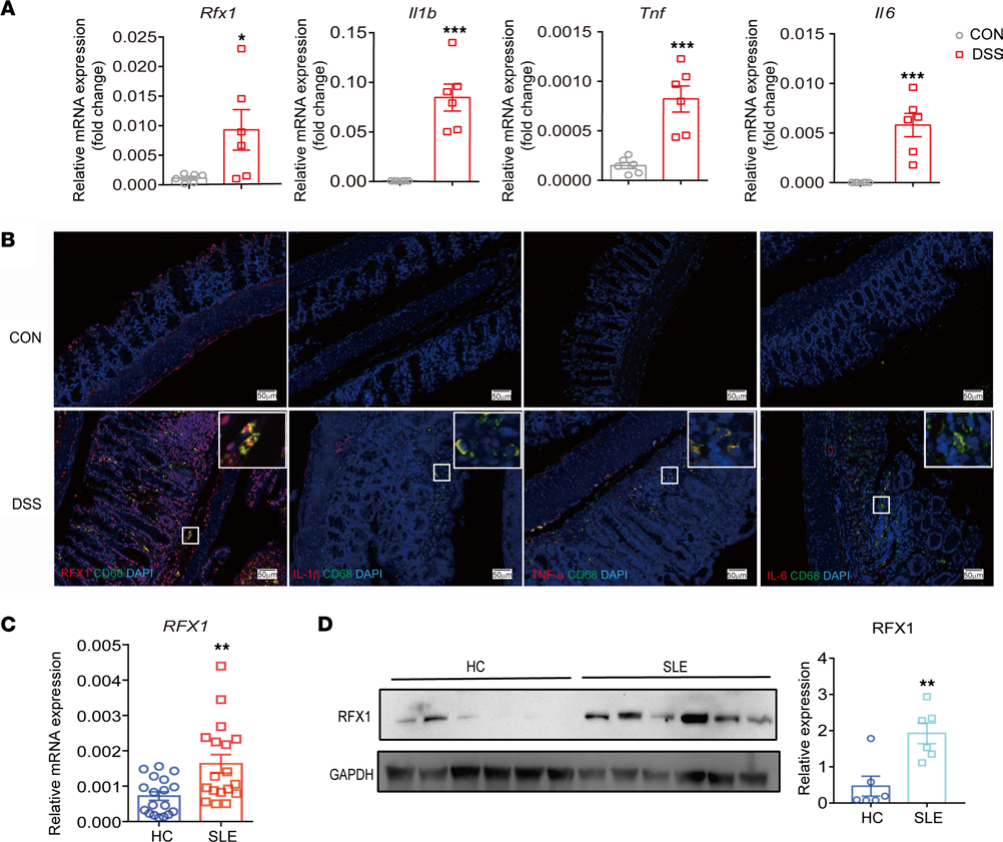
RFX1 expression was increased in inflammatory diseases. (**A**) The relative mRNA expression of *Rfx1*, *Il1b*, *Tnf*, and *Il6* in colon from control (NC) and colitis (DSS) mice (*n* = 6 each group). (**B**) Representative images of IF staining of indicated positive cells in colon from control (NC) or colitis (DSS) mice. Scale bar: 50 mm. (**C**) The relative mRNA expression of RFX1 in CD14^+^ monocyte from healthy controls (HC) and patients with SLE (*n* = 18 each group). (**D**) Western blot was used to detect the protein expression of RFX1 in CD14^+^ monocytes from HC and patients with SLE (*n* = 6 each group). The protein expression was normalized to GAPDH. Data represent mean ± SEM, 2-tailed Student’s *t* test. *P < 0.05, **P < 0.01, ***P < 0.001.

M1 macrophages play an important role in the pathogenesis of inflammatory and autoimmune diseases. Therefore, we constructed the dextran sulfate sodium–induced (DSS-induced) mice with colitis to detect the RFX1 expression in the inflammatory colon tissue (Supplemental Figure 1, A–C; supplemental material available online with this article; https://doi.org/10.1172/jci.insight.165546DS1). We found that the relative mRNA expression of *Rfx1* and M1 macrophage–related genes *Il6*, *Tnf*, and *Il1b* were significantly increased in the colon tissue of mice with colitis compared with the control group (Figure 1A). The expressions of RFZ1, IL-6, TNF-α, and IL-1β were also increased in CD68^+^ macrophage detected by IF staining in the colon from mice with colitis (Figure 1B). Moreover, we found that the relative mRNA and protein expressions of RFX1 in CD14^+^ monocytes from the peripheral blood of patients with SLE were significantly increased compared with healthy controls (HC) (Figure 1, C and D). To explore the expression pattern of RFX1 in macrophages of autoimmune diseases, we analyzed the single-cell RNA-Seq data of skin lesions from patients with SLE (GSE179633). Dermal macrophages identified by expression of markers such as AIF1, LYZ, CD68, MRC1, and CD163, formed 2 clusters (Cluster 0 and Cluster 1) (Supplemental Figure 2A). Cluster 1 expressed higher levels of CCL15, CCR7, CD40, IDO1, IL-1β, KYNU, CD80, and IL-6 compared with Cluster 0, suggesting a M1-like phenotype. The percentage of Cluster 1 with higher expression levels of *RFX1*, *IL6*, *TNF*, and *IL1B* was increased in patients with SLE compared with HC (Supplemental Figure 2, B–D), which suggested the RFX1 expression may be associated with the inflammatory cell cluster. Considering the positive effect of M1 macrophages on the tumor, we analyzed the expression profiles of *RFX1*, *IL6*, *TNF*, and *IL1B* according to Gene Expression Profiling Interactive Analysis (GEPIA) (23), which is a web server for analyzing the RNA-Seq expression data from the TCGA and the GTEx projects, we found the expression levels of *RFX1*, *IL6*, *TNF*, and *IL1B* were all increased in multiple tumor tissues including tissues from patients with ovarian cancer (OA), esophageal cancer (ESCA), glioblastoma (GBM), pancreatic cancer (PAAD), and stomach cancer (STAD) (Supplemental Figure 3), consistent with the results of inflammatory and autoimmune diseases. The RNA-Seq data sets used are based on the UCSC Xena project (http://xena.ucsc.edu), which are computed by a standard pipeline. The tumor data sets matched TCGA normal and GTEx data.

### RFX1 expression was upregulated in M1 macrophage.

**Figure 2:**
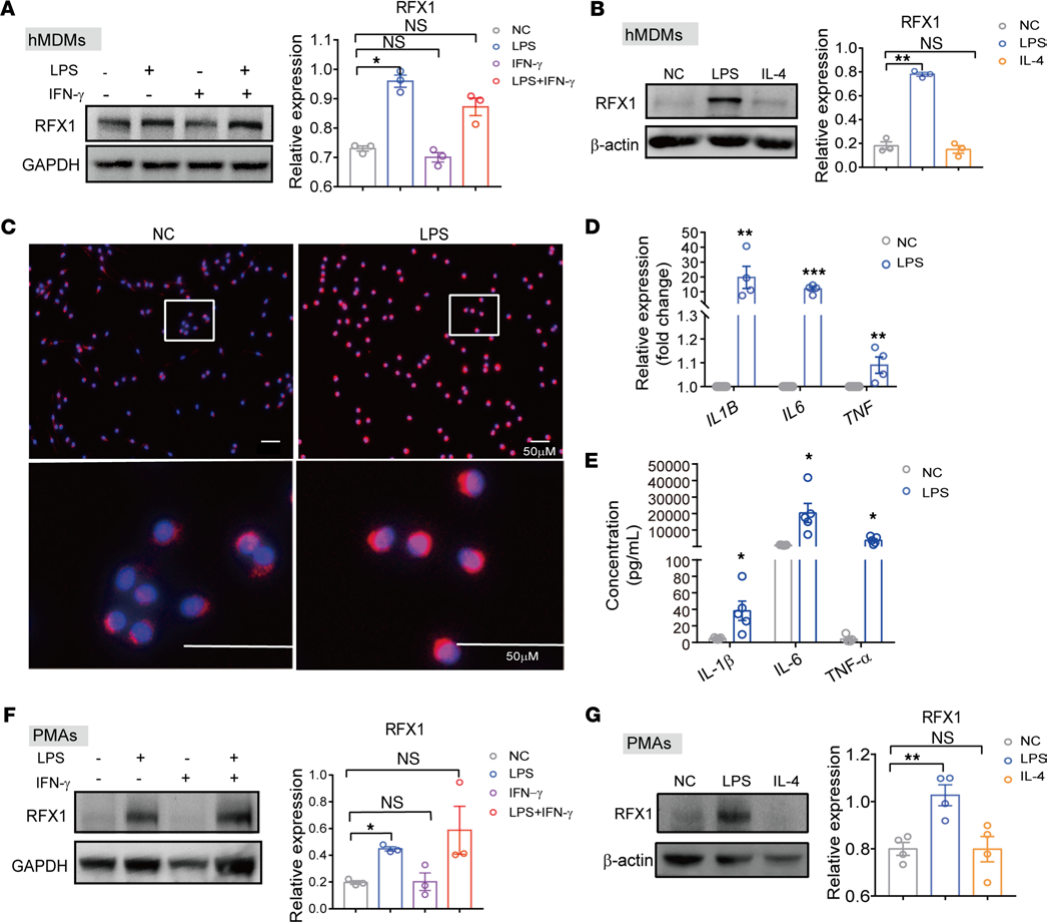
The RFX1 expression in macrophage polarization. (**A**) The protein expression of RFX1 in hMDMs treated with LPS (1 μg/mL), IFN-γ (20 ng/mL), or both (*n* = 3). (**B**) The protein expression of RFX1 in hMDMs treated with LPS or IL-4 (20 ng/mL) (*n* = 3). (**C**) The hMDMs with or without LPS treatment were immunostained with RFX1 antibody (red) and DAPI (blue). Scale bar: 50 μM. (**D**) The relative mRNA expressions of *TNF*, *IL6*, and *IL1B* in hMDMs with or without LPS stimulation (*n* = 4). (**E**) The concentrations of TNF-α, IL-6, and IL-1β in culture supernatants from hMDMs with or without LPS stimulation (*n* = 5). (**F**) The protein expression of RFX1 in PMAs treated with LPS, IFN-γ, or both (*n* = 3). (**G**) The protein expression of RFX1 in PMAs treated with LPS or IL-4 (*n* = 4). The data of protein expression were expressed after being normalized by GAPDH (**A** and **F**) or β-actin (**B** and **G**). Data represent mean ± SEM. One-way ANOVA with Dunnett’s multiple-comparison test was used in **A**, **B**, **F**, and **G**. Two-tailed Student’s *t* test was used in **D** and **E**. *P < 0.05, **P < 0.01, ***P < 0.001.

To detect the expression of RFX1 in M1 macrophages, we stimulated human monocyte-derived macrophages (hMDMs) with LPS and IFN-γ separately or simultaneously. The human CD14^+^ monocytes were separated from peripheral blood mononuclear cells (PBMC) and were induced by macrophage CSF (M-CSF) (50 ng/mL) to differentiate into hMDMs in vitro. Compared with the control group, the relative protein expression of RFX1 was significantly increased in LPS-stimulated macrophages but not in LPS plus IFN-γ or only IFN-γ–stimulated macrophages (Figure 2A). To further explore whether RFX1 expression was also related to M2 polarization, we detect the expression level of RFX1 in LPS or IL-4–stimulated hMDMs. Unlike LPS, IL-4 administration could not alter the relatively RFX1 protein expressions in hMDMs (Figure 2B). Immunofluorescence (IF) staining showed that RFX1 in the nucleus was increased in LPS-induced hMDMs (Figure 2C). In addition, we also verified that LPS treatment significantly increased the mRNA and protein expression levels of proinflammatory cytokines IL-6, TNF-α, and IL-1β in hMDMs (Figure 2, D and E). Furthermore, the RFX1 expression was also detected in mouse peritoneal macrophages (PMAs). Compared with the control group, the relative protein expression of RFX1 was significantly increased in LPS and LPS plus IFN-γ–stimulated PMAs but not in IFN-γ or IL-4–stimulated PMAs (Figure 2, F and G). These results indicate that RFX1 might be involved in LPS-induced M1 macrophage polarization.

### RFX1 promoted M1 macrophage polarization in vitro.

**Figure 3:**
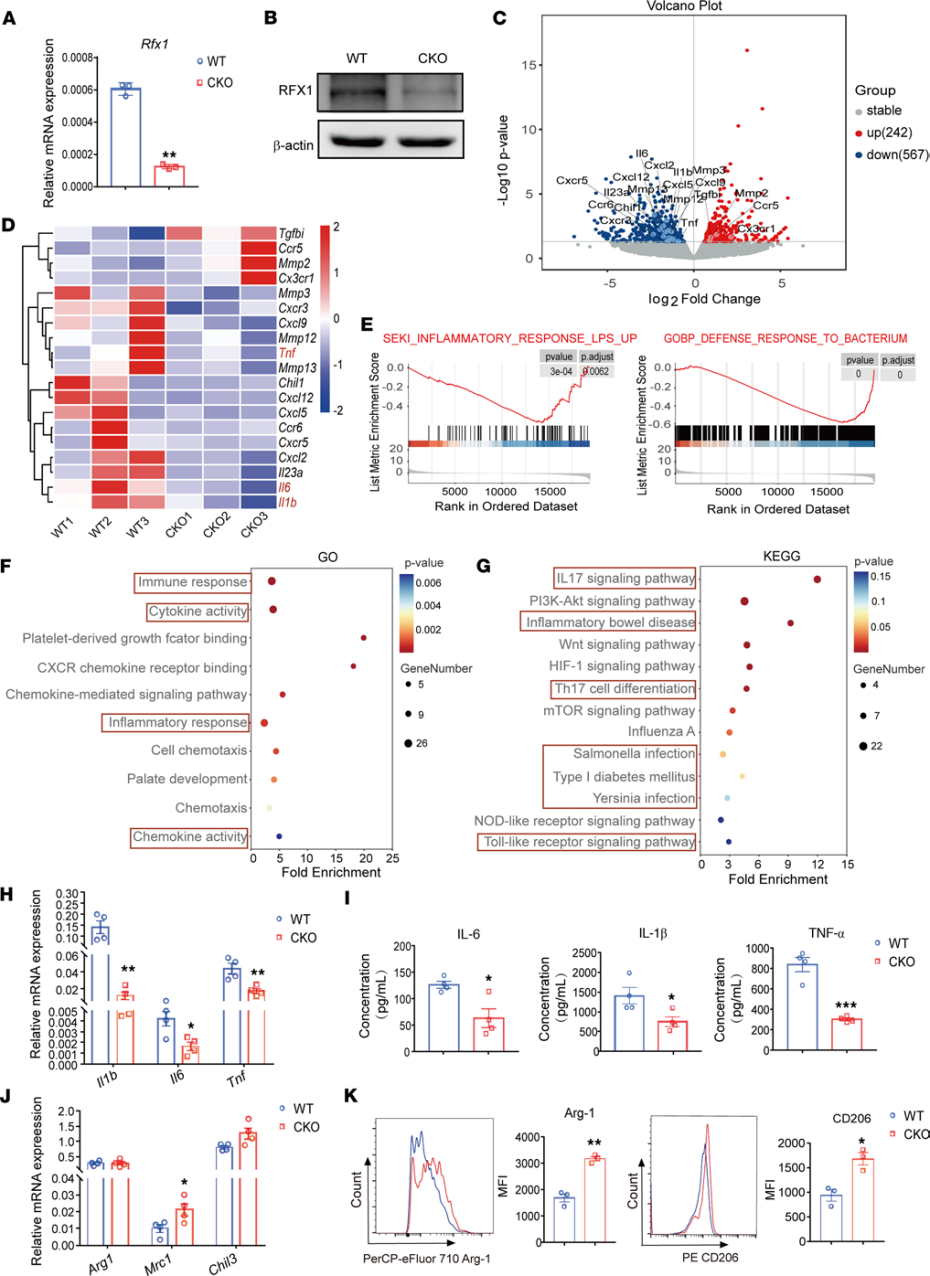
*Rfx1* KO inhibited M1 macrophage polarization. (**A** and **B**) The mRNA (*n* = 3 each group) (**A**) and protein (**B**) expressions of RFX1 in PMAs from *Rfx1*^fl/fl^ (WT) and *Rfx1*^fl/fl^Lyz2-Cre (CKO) mice, respectively. (**C**) Volcano plot of differently expressed genes between LPS-induced M1 PMAs from WT or CKO mice (*n* = 3 each group). (**D**) Heatmap revealed differentially expressed macrophage polarization–related genes between M1 PMAs from WT and CKO mice. (**E**) Enrichment analysis of differentially expressed genes in WT or CKO group based on GSEA pathway database. (**F** and **G**) Significantly enriched GO terms (**F**) and KEGG pathways (**G**) of downregulated genes in M1 PMAs from CKO mice. |Log_2_FC|≥1 and *P* ≤ 0.05 are the screening criteria for differential genes detected by RNA-Seq between 2 groups. (**H** and **I**) The relative mRNA (**H**) and protein expressions in culture supernatant (**I**) of M1-related genes *Tnf*, *Il6*, and *Il1b* were detected by qPCR and ELISA (*n* = 4 each group). (**J**) The relative expression of the indicated M2-related genes in M1 PMAs from WT and CKO mice (*n* = 4 each group). (**K**) Representative flow cytometry histograms and mean fluorescence intensities (MFI) statistics of Arg-1 and CD206 (*n* = 3 each group). Data represent mean ± SEM, using 2-tailed Student’s *t* test for **A** and **H**–**K**. *P < 0.05, **P < 0.01, ***P < 0.001.

To investigate the function of RFX1 in macrophage polarization, we constructed the Rfx1^fl/fl^Lyz2-Cre conditional KO (CKO) mice to inhibit RFX1 expression specifically in macrophages; the littermate Rfx1^fl/fl^ mice were used for WT controls. Compared with PMAs from WT mice, the relative expressions of RFX1 mRNA and protein were significantly reduced (Figure 3, A and B) in PMAs from CKO mice (CKO PMAs). Moreover, we performed RNA-Seq to detect the effect of RFX1 KO on macrophage polarization in LPS-stimulated PMAs from WT and CKO mice. In total, 242 upregulated and 567 downregulated genes were identified between CKO and WT mice (Figure 3C). Some M1-related genes such as *Tnf*, *Il6*, and *Il1b* were identified in the downregulated genes (Figure 3D). Gene set enrichment analysis (GSEA) shows that the downregulated genes in CKO PMAs were enriched in several M1-related pathways, including defense response to bacterium and LPS-stimulated inflammatory response (Figure 3E). The Gene Ontology (GO) analysis suggested that the downregulated genes in CKO PMAs were associated with immune and inflammatory response, the activities of cytokine and chemotaxis, and positive regulation of inflammatory response (Figure 3F). Besides, the Kyoto Encyclopedia of Genes and Genomes (KEGG) pathway analysis showed that the downregulated genes in CKO PMAs were enriched in multiple inflammatory signaling pathways, including IL-17A and TLR, Th17 cell differentiation, M1-related bacterial infection, and autoimmunity diseases like IBD and type 1 diabetes mellitus (Figure 3G).

In addition, we also validated the effect of RFX1 on macrophage M1 polarization. Compared with WT PMAs, the relative mRNA expression levels of M1-related cytokine genes *Il6*, *Tnf*, and *Il1b* were significantly reduced in CKO PMAs stimulated with LPS (Figure 3H). The protein concentrations of IL-6, TNF-α, and IL-1β in supernatant from CKO PMAs were also decreased significantly (Figure 3I). In contrast, the mRNA expression of the M2-related gene *Mrc1* was upregulated, and the protein levels of M2-related genes Arg-1 and CD206 were increased significantly in LPS-treated CKO PMAs according to the mean fluorescence intensities (MFI) of flow cytometry detection (Figure 3, J and K). However, the relative mRNA and protein expression of IL-10 was not altered in M1 PMAs with Rfx1 knockdown (Supplemental Figure 4, A and B). To determine the proinflammatory or antiinflammatory capacity of macrophages with different RFX1 expressions, we detected the phagocytosis capacity of M1 PMAs infected with pLV-NC and pLV-shRfx1 for fluorescent particles. We found the phagocytosis capacity of macrophages with decreased RFX1 expression was significantly inhibited (Supplemental Figure 4C); the culture supernatant from M1 PMAs infected with pLV-shRfx1 inhibited the cytotoxic T (CD107a^+^CD4^+^) cell differentiation but not Th1 (IFN-γ^+^CD4^+^) or Th2 (IL4^+^CD4^+^) differentiation, which suggested that Rfx1 knockdown in macrophage increased T cell suppression (Supplemental Figure 4, D and E).

**Figure 4:**
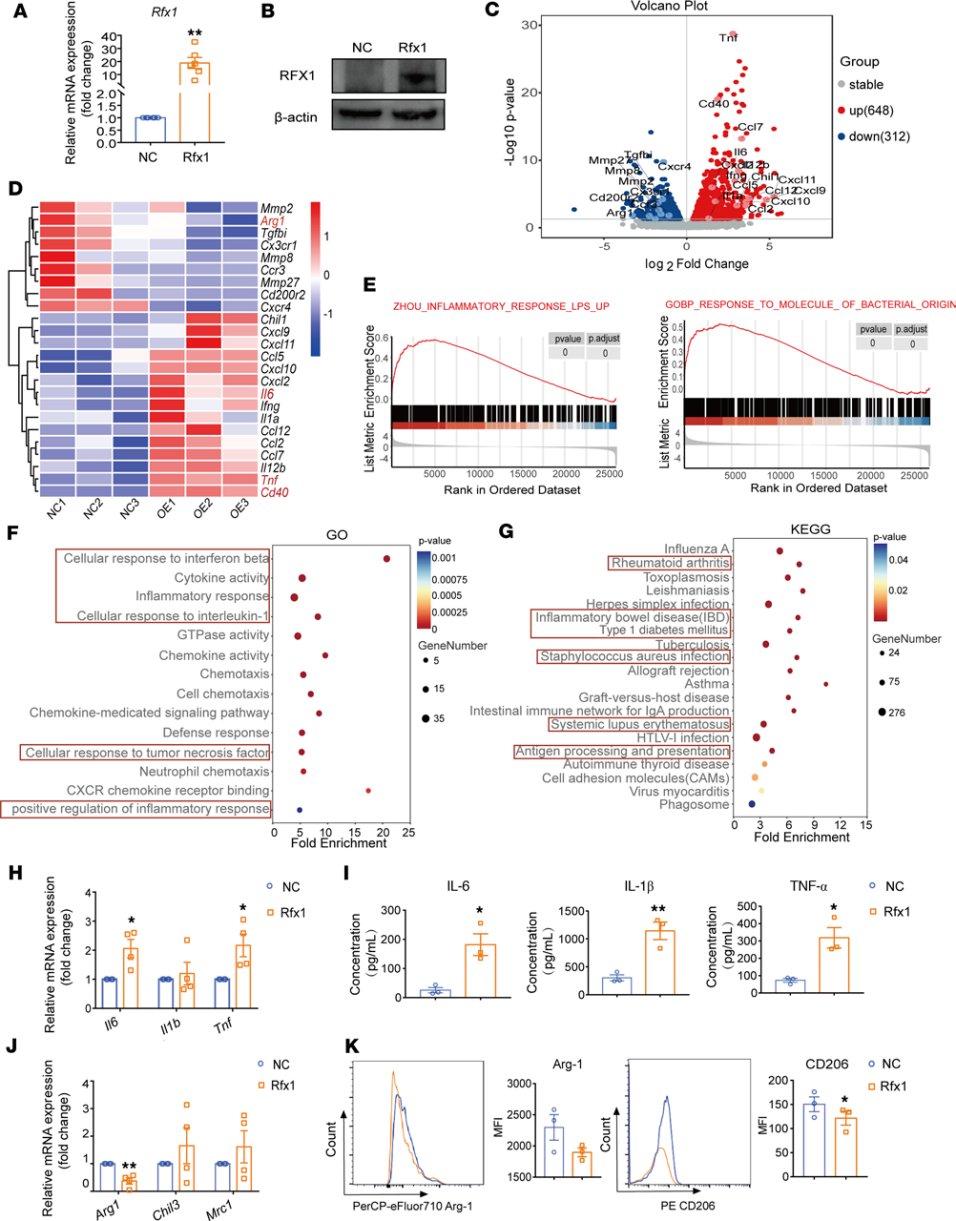
RFX1 overexpression promoted M1 macrophage polarization. (**A** and **B**) The relative mRNA (*n* = 6) (**A**) and protein (**B**) expressions of RFX1 in LPS-induced M1 PMAs infected with pLV-NC and pLV-Rfx1. (**C**) Volcano plot of differently expressed genes between M1 PMAs transfected with pLV-NC and pLV-Rfx1 (*n* = 3). (**D**) Heatmap indicated differentially expressed macrophage polarization–related genes between M1 PMAs infected with pLV-NC and pLV-Rfx1. (**E**) GSEA pathway database was used to detect enrichment plots of differently expressed genes in each group. (**F** and **G**) Significantly enriched GO terms (**F**) and KEGG pathways (**G**) in M1 PMAs infected with pLV-Rfx1. |Log_2_FC|≥1 and *P* ≤ 0.05 are the screening criteria for differential genes detected by RNA-Seq between 2 groups. (**H** and **I**) The relative mRNA expressions (**H**) (*n* = 4) and the protein concentrations (**I**) (*n* = 3) in culture supernatant of TNF-α, IL-6, and IL-1β in PMAs were detected. (**J**) The relative mRNA levels of the indicated M2-related genes in M1 PMAs infected with pLV-NC and pLV-Rfx1 (*n* = 4). (**K**) Representative flow cytometry histograms and MFI statistics of Arg-1 and CD206 in each group (*n* = 3). Data represent mean ± SEM; 2-tailed Student’s *t* test was used for **A** and **H**–**K**. *P < 0.05, **P < 0.01.

Furthermore, we overexpressed Rfx1 in PMAs by lentivirus vector pLV-Rfx1 infection. The results of quantitative PCR (qPCR) and Western blot showed a significantly increased expression of Rfx1 in pLV-Rfx1–infected PMAs compared with negative control (NC) (Figure 4, A and B). The expression of 648 genes was increased and the expression of 312 genes was reduced was reduced in Rfx1-overexpressed PMAs with LPS stimulation compared with the NC group (Figure 4C), among which the expression of M1-related genes such as *Tnf*, *Il6*, *Cd40*, and *Il12b* was increased and M2-related gene expression such as *Arg1* was reduced in Rfx1-overexpressed M1 PMAs (Figure 4D). These upregulated genes in Rfx1-overexpressed PMAs were enriched in the defensive response to bacterium and LPS-stimulated inflammatory response pathways by GSEA (Figure 4E). The increased genes in Rfx1-overexpressed macrophage were associated with multiple inflammatory responses such as IFN-β, TNF-α, and IL-1β and the activity of cytokine and chemotaxis according to GO analysis (Figure 4F). The KEGG pathway indicated that the increased genes were enriched in various autoimmune diseases, including rheumatoid arthritis, IBD, SLE, and bacterial infections (Figure 4G). Moreover, the relative mRNA expression levels of IL-6 and TNF-α but not IL-1β were significantly increased (Figure 4H), and the protein concentrations in the culture supernatant of TNF-α, IL-6, and IL-1β in M1 PMAs infected with pLV-Rfx1 were also increased significantly (Figure 4I). The mRNA expression of Arg-1 was inhibited in PMAs infected with pLV-Rfx1 (Figure 4J). However, the MFI of CD206 but not Arg-1 was significantly reduced in Rfx1-overexpressed M1 PMAs (Figure 4K). The relative mRNA and protein expression of IL-10 was not altered after RFX1 overexpression in M1 PMAs (Supplemental Figure 4, F and G). However, we found that the phagocytosis capacity of macrophages with RFX1 overexpression was significantly increased (Supplemental Figure 4H), and the culture supernatant from M1 PMAs infected with pLV-Rfx1 promoted the cytotoxic T cell differentiation and inhibited Th2 differentiation (Supplemental Figure 4I).

The effect of RFX1 overexpression was also observed in LPS-induced M1 hMDMs. Compared with the NC group, the mRNA expression levels of *RFX1*, *IL1*Β, *TNF*, *CCL2*, and *TLR4* were significantly increased in hMDMs infected with pLV-RFX1 (Supplemental Figure 5, A and B). The RNA-Seq was used to detect the differentially expressed genes in hMDMs with RFX1 overexpression and NC group. The GO analysis suggested that the increased genes in hMDMs with RFX1 overexpression were closely related to inflammatory response and inflammation response increased by LPS (Supplemental Figure 5, C and D). The increased genes were enriched in response to the type I IFN signaling pathway, which played a vital role in autoimmune diseases. The GSEA also indicated that the increased genes in hMDMs with RFX1 overexpression were significantly correlated with the inflammatory response (Supplemental Figure 5E).

### RFX1 KO alleviated DSS-induced colitis and promoted tumor development.

**Figure 5:**
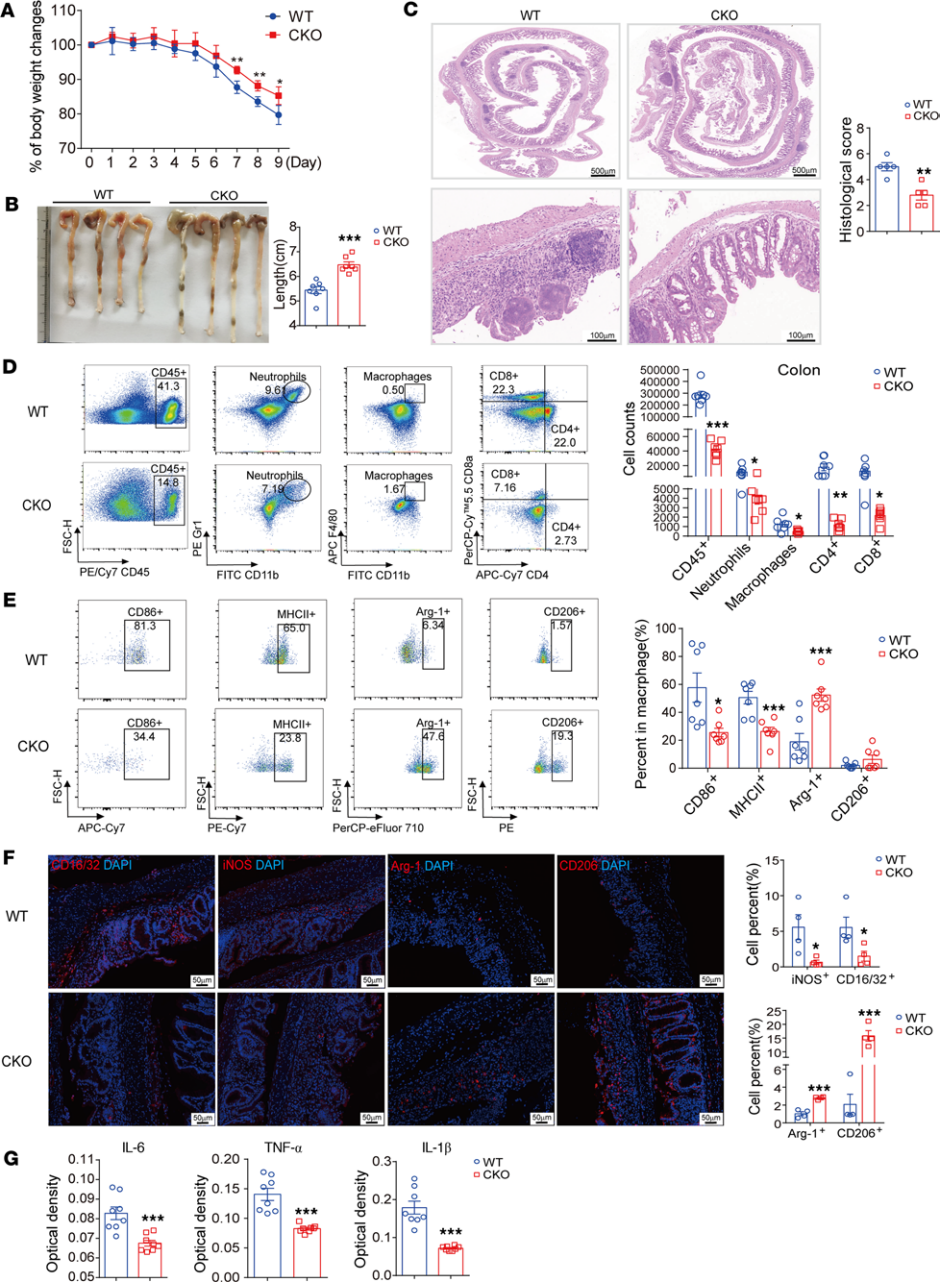
*Rfx1* KO extenuated DSS-induced colitis in mice. (**A**) Daily weight change of WT and CKO mice with colitis (*n* = 7 per group). (**B**) Representative image of colon from each group and measured length of the colon (*n* = 7 per group). (**C**) Representative images of H&E staining and histological score of the colons from WT and CKO mice with colitis (*n* = 5 per group). Scale bar: 500 mm (top), 100 mm (bottom). (**D**) Cells were isolated from the colon and identified the amounts of indicated immune cell infiltrated in colon from WT and CKO mice (*n* = 7 per group). (**E**) The proportions of CD86^+^, MHCII^+^, Arg-1^+^, or CD206^+^ cells in macrophages from the colon were displayed (*n* = 7 per group). (**F**) Representative images of IF staining and percentages of indicated positive cells in the colon (*n* = 4 per group). Scale bar: 50 mm. (**G**) The protein expressions of TNF-α, IL-6, and IL-1β in serum were measured (*n* = 8 per group). Data represent mean ± SEM. Two-tailed Student’s *t* test was used. *P < 0.05, **P < 0.01, ***P < 0.001.

First, we detected the effect of Rfx1 deficiency on the intestinal and spleen tissues of mice under normal conditions. We found that CKO mice did not exhibit spontaneous intestinal inflammatory injury and spleen size change (Supplemental Figure 6, A–C). For detecting the effect of Rfx1 on M1 macrophage polarization in vivo, we induced WT and CKO mice with colitis induced by DSS. Compared with WT mice, the weight loss and colon shortening in CKO mice were significantly alleviated (Figure 5, A and B). The infiltration of immune cells was significantly reduced, and the tissue damage to epithelial crypts and villi was significantly ameliorated in the colon of CKO mice, as indicated by H&E staining (Figure 5C). The proportions of CD45^+^ cells, CD4^+^ T cells, and macrophages were also significantly reduced in PBMCs from CKO mice (Supplemental Figure 6D). Furthermore, the infiltrated CD45^+^ cells, neutrophils, macrophage, CD4^+^ T cells, and CD8^+^ T cells were significantly decreased in the colon of CKO mice (Figure 5D). We also determined the expression of macrophage polarization–related molecules in infiltrated colonic macrophages from WT and CKO mice with colitis (Figure 5E). The proportions of CD86^+^ and MHCII^+^ macrophages (M1) were lessened, while the proportion of Arg-1^+^ macrophage (M2) was significantly increased in macrophages of CKO mice. Moreover, the IHC staining showed that the percentages of CD16/32^+^ and iNOS^+^ cells were decreased, while the percentages of Arg-1^+^ and CD206^+^ cells were increased significantly in the colon of CKO mice (Figure 5F). In addition, the expression levels of IL-6, IL-1β, and TNF-α in serum from CKO mice were lower than those in WT mice (Figure 5G). These results suggest that the KO of Rfx1 in myeloid cells inhibited the inflammatory macrophage activation in vivo and alleviated intestinal injury in DSS-induced mice with colitis.

M1 macrophages are tumor resistant due to intrinsic phagocytosis and the enhanced antitumor inflammatory reaction. To determine whether RFX1 KO in macrophages influenced tumor development, we performed the s.c. tumor xenograft model in CKO mice. In total, 2 × 10^5^ murine melanoma cell line B16F10 cells in 100 μL sterile PBS were injected s.c. into the shaved right flank of anesthetized WT or CKO mice. Compared with WT mice, the tumor size in CKO mice was significantly enlarged 6–12 days after tumor cell injection (Supplemental Figure 7, A and B). The expression of Ki67 in tumors from CKO mice was significantly augmented, revealing the increased proliferation in tumors from CKO mice (Supplemental Figure 7C). Furthermore, the percentages of CD45^+^ immune cells expressing antiinflammatory cytokines IL-10 or IL-13 were increased, while the percentage of CD45^+^ cells expressing IL-23 was decreased (Supplemental Figure 7D). In addition, the percentages of CD86^+^ and MHCII^+^ macrophage infiltrated in CKO mice were decreased, while CD206^+^ macrophage was increased significantly (Supplemental Figure 7E). The IF staining showed that the proportions of Arg-1^+^ cells and CD206^+^ cells were also enlarged in tumor tissues from CKO mice (Supplemental Figure 7F). These results indicate that Rfx1 deficiency restrained proinflammatory macrophage polarization and promoted melanoma development.

### Rfx1 KO mitigated the pathogenesis of imiquimod-induced (IMQ-induced) lupus-like mice.

**Figure 6:**
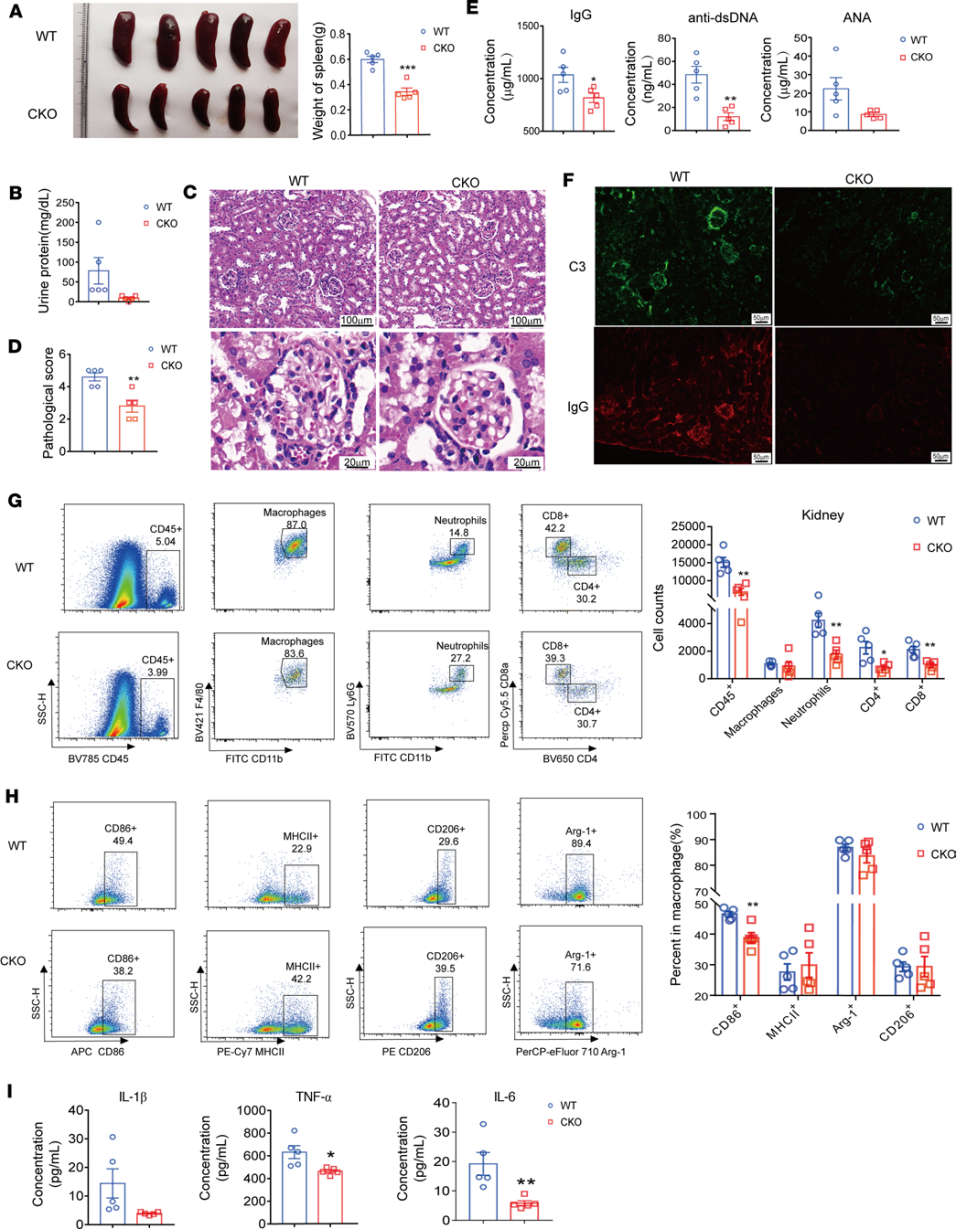
*Rfx1* KO mitigated pathogenesis of IMQ-induced lupus-like mice. Each group contained 5 mice. (**A**) The images of spleen from each group and measured spleen weight. (**B**) The urine protein concentrations of lupus-like WT and CKO mice. (**C**) Representative H&E staining of kidney. Scale bar: 100 mm (top), 20 mm (bottom). (**D**) Kidney pathological score in samples isolated from lupus-like mice was evaluated in a blinded manner. (**E**) The concentrations of IgG, anti-dsDNA antibodies, and ANA in serum were measured. (**F**) Representative IF staining of the kidneys of WT and CKO mice. Scale bar: 50 mm. (**G**) Representative gating and the infiltrated amount of indicated immune cells in kidney from IMQ-induced WT and CKO mice. (**H**) Representative gating and the proportions of CD86^+^, MHCII^+^, Arg-1^+^, or CD206^+^ cells in macrophages from kidney were displayed. (**I**) The concentrations of TNF-α, IL-6, and IL-1β in serum were measured. Data represent mean ± SEM. Two-tailed Student’s *t* test was used. *P < 0.05, **P < 0.01, ***P < 0.001.

We next investigated the role of RFX1 deficiency in the macrophage of IMQ-induced lupus-like mice. The TLR7 agonist, IMQ, induced lupus-like mice that were prepared as previously described (24). Compared with WT mice, splenomegaly was improved, and the concentration of urine protein was decreased significantly in CKO mice (Figure 6, A and B). The infiltration of immune cells in the glomerulus and renal pathology scores were alleviated in CKO mice (Figure 6, C and D). Moreover, the concentrations of anti-dsDNA antibody and total IgG antibody but not antinuclear antibody (ANA) in serum were decreased significantly in CKO mice (Figure 6E). The depositions of complement 3 (C3) and IgG in kidney tissues from CKO mice were significantly decreased as well (Figure 6F). In addition, the infiltrations of total immune cells (CD45^+^), neutrophils (CD11b^+^Gr1^+^), CD4^+^, and CD8^+^ cells were significantly reduced in the kidney from CKO mice (Figure 6G). Furthermore, the expression of CD86 was reduced in renal macrophages from CKO mice (Figure 6H). The protein levels of cytokines IL-6 and TNF-α but not IL-1β were lessened significantly in serum from CKO mice (Figure 6I). These results suggest that RFX1 was essential for M1 macrophage–mediated inflammatory response in IMQ-induced lupus-like mice and that RFX1 deficiency could improve lupus development.

### RFX1 directly regulated APOBEC3A transcription.

**Figure 7:**
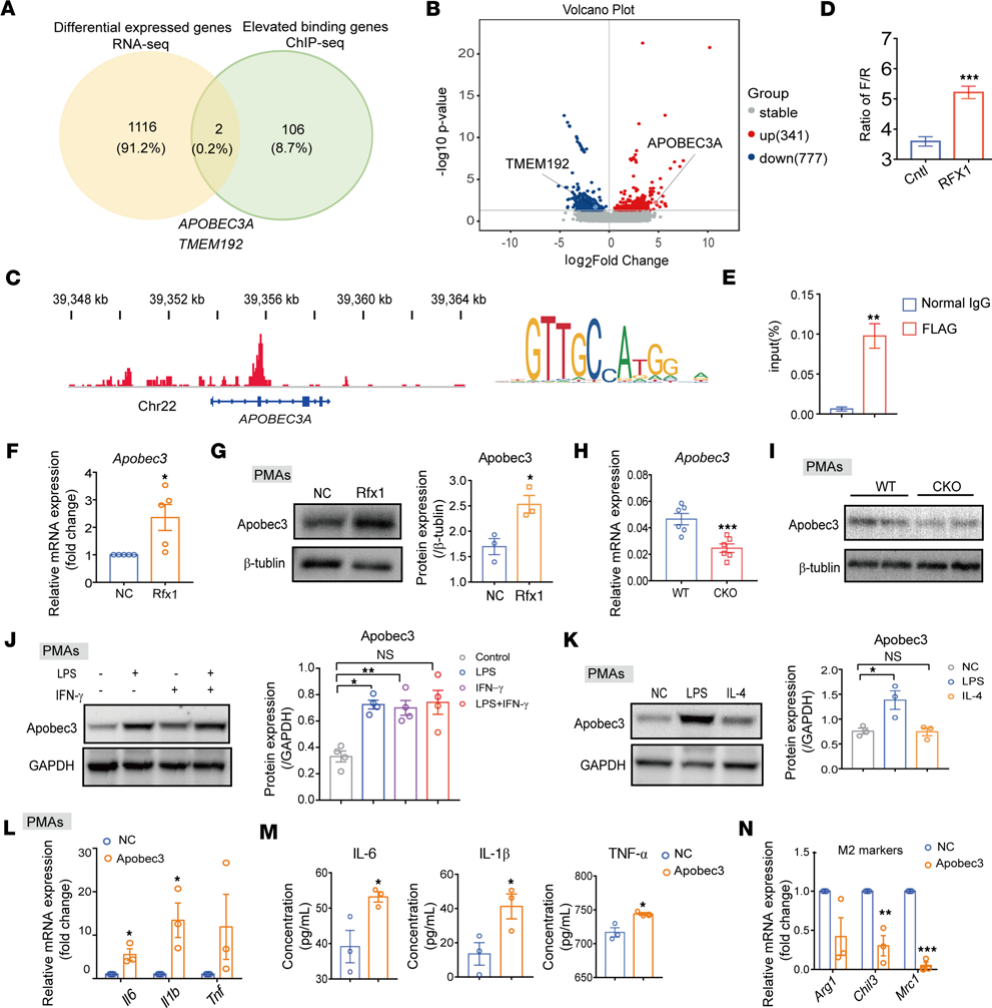
RFX1 regulated macrophage polarization by APOBEC3A. (**A**) Venn diagram showing 2 overlapping genes between the differently expressed genes detected by RNA-Seq and the potential target genes of RFX1 determined by ChIP-Seq. (**B**) Volcano plot of differentially expressed genes between control and RFX1-overexpressed M1 hMDMs (*n* = 3). (**C**) The ChIP-Seq profile of RFX1 in the *APOBEC3A* gene locus and sequence logo of the RFX1 motif were indicated. (**D**) The relative expression of luciferase in each group was determined by a luciferase reporter assay containing the genomic fragment of *APOBEC3A* in 293T cells (*n* = 5). (**E**) The ChIP-qPCR was performed on 293T cells infected with pLV-RFX1 using anti-FLAG antibodies (*n* = 3). (**F** and **G**) The relative mRNA (*n* = 5) (**F**) and protein (*n* = 3) (**G**) expressions of Apobec3 in PMAs were analyzed. The protein expression was shown after being normalized β-tublin. (**H** and **I**) The relative mRNA (*n* = 6 per group) (**H**) and protein (*n* = 2 per group) (**I**) expressions of Apobec3 in M1 PMAs from WT and CKO mice were determined. (**J** and **K**) Western blot was used to determine the protein expressions of Apobec3 in PMAs with indicated treatment (**J**, *n* = 4; **K**, *n* = 3). The protein expressions were shown after being normalized to GAPDH. (**L** and **M**) The mRNA (**L**) and protein (**M**) expressions of *Il6*, *Il1b*, and *Tnf* in PMAs infected with pLV-NC and pLV-Apobec3 (*n* = 3). (**N**) The mRNA expressions of *Arg1*, *Chils*, and *Mrc1* in PMAs infected with pLV-NC and pLV-Apobec3 (*n* = 3). Data represent mean ± SEM. One-way ANOVA with Dunnett’s multiple-comparison test was used in **J** and **K**. Two-tailed Student’s *t* test was used in **D**–**H** and **L**–**N**. *P < 0.05, **P < 0.01, ***P < 0.001.

RFX1, as a transcription factor, regulates target gene transcription by binding their promoter or enhancer element. To identify the target genes of RFX1 in regulating M1 macrophage polarization, we performed ChIP-Seq and RNA-Seq in LPS-induced hMDMs with or without RFX1 overexpression. We identified 106 genes surrounded by the potentially increased RFX1 binding peaks and 1,116 differentially expressed genes in hMDMs infected with pLV-RFX1 compared with the NC group. Combining the data from ChIP-Seq and RNA-Seq, we found 2 common genes between the differentially expressed genes and the binding fragment–related genes, *APOBEC3A* and *TMEM192* (Figure 7A). As shown in the volcano plot, the expression of *APOBEC3A* was increased, while *TMEM192* expression was decreased in RFX1-overexpressed hMDMs stimulated with LPS (Figure 7B).

The previous studies found that APOBEC3A promoted proinflammatory M1 but inhibited M2 macrophage polarization and was abnormally expressed in renal biopsy tissue and PBMC from patients with SLE (25, 26). Therefore, we predicted that APOBEC3A might be a direct target of RFX1 to induce M1 macrophage polarization. As shown in ideal graph visualizer (IGV), high RFX1 enrichment was observed at the *APOBEC3A* locus with predicted binding sites of RFX1 in hMDMs treated with LPS (Figure 7C). We then constructed a dual-luciferase reporter gene system to detect the regulatory activity of the enhancer region with the predicted RFX1 binding sites. The results show that overexpression of RFX1 increased the transcription activity of the enhancer region with RFX1 binding sites of *APOBECEA* (Figure 7D). Furthermore, the ChIP-qPCR assay also confirmed that RFX1 could directly bind to the enhancer region of the *APOBEC3A* gene (Figure 7E). As we expected, the APOBEC3A/Apobec3 expression was augmented in RFX1-overexpressed PMAs (Figure 7, F and G) and hMDMs (Supplemental Figure 8A). In PMAs from Rfx1 CKO mice, the Apobec3 expression was decreased compared with the WT control (Figure 7, H and I). Furthermore, the relative expression levels of Apobec3 were increased in PMAs in the presence of LPS stimulation but not in the presence of IL-4 (Figure 7, J and K). We also found that LPS significantly increased APOBEC3A expression in hMDMs (Supplemental Figure 8B).

Furthermore, we determined the role of Apobec3 in M1 macrophage polarization. We found that the relative mRNA expressions of IL-6 and IL-1β but not TNF-α were significantly promoted, and the concentrations of these cytokines in cell culture supernatants were significantly increased in Apobec3-overexpressed PMAs compared with the control group (Figure 7, L and M). In addition, overexpressed Apobec3 inhibited the relative mRNA expression of M2-related genes *Chil3* and *Mrc1* but did not alter the expression of IL-10 in PMAs (Figure 7N and Supplemental Figure 8, C and D). Consistent with those in mice, overexpression of APOBEC3A in hMDMs increased the relative mRNA expression of IL-6 and IL-1β (Supplemental Figure 8E). Taken together, our results indicate that RFX1 promoted the proinflammatory macrophage polarization by binding the enhancer region of the *APOBEC3A* gene to promote APOBEC3A expression. To further confirm that RFX1 regulates macrophage inflammation by APOBEC3A, we overexpressed RFX1 expression in hMDMs after APOBEC3A knockdown and detected the level of macrophage activation. These results show that overexpression of RFX1 in hMDMs interfering with APOBEC3A expression had no significant effect on the expressions of TNF-α, IL-6, IL-1β, and IL-10 (Supplemental Figure 8, F–J).

### APOBEC3A regulates IL-6 and TNF-α expression by inducing demethylation.

**Figure 8:**
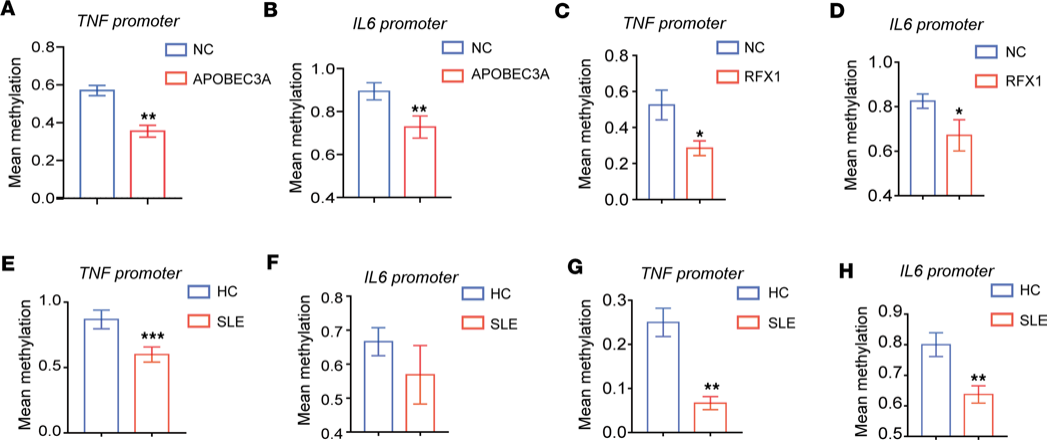
APOBEC3A and RFX1 regulated the methylation of M1-related genes *TNF* and *IL6*. (**A** and **B**) The mean methylation levels of CpG sites within *TNF* (**A**) and *IL6* (**B**) promoters in hMDMs infected with pLV-NC or pLV-APOBEC3A (*n* = 4 per group). (**C** and **D**) The mean methylation levels of CpG sites within *TNF* (**C**) and *IL6* (**D**) promoters in M1 hMDMs infected with pLV-NC or pLV-RFX1 (*n* = 4 per group). (**E** and **F**) The mean methylation levels of CpG sites within *TNF* (**E**) and *IL6* (**F**) promoters in CD14^+^ monocytes from HC or patients with SLE (*n* = 5 per group). (**G** and **H**) The mean methylation levels of CpG sites within *TNF* (**G**) and *IL6* (**H**) in M1 hMDMs from HC or patients with SLE (*n* = 6 per group). Ten respective clones from each group were sequenced. Data represent mean ± SEM, using 2-tailed Student’s *t* test. *P < 0.05, **P < 0.01, ***P < 0.001.

Considering that the effect of APOBEC3A on deamination of methylcytosine (mC) (27), we further determined the methylation of *IL6*, *TNF*, and *IL1B* promoters in APOBEC3A-overexpressed hMDMs. Compared with the control, the mean methylation levels of CpGs within *IL6* and *TNF* promoters were significantly decreased in hMDMs with APOBEC3A overexpression (Figure 8, A and B, and Supplemental Figure 9, A and B). However, the mean methylation level of CpGs within *IL1B* promoter was unaltered (Supplemental Figure 9C). Furthermore, we also examined the mean methylation levels of CpGs within *IL6*, *TNF*, and *IL1B* promoters in hMDMs with or without LPS treatment (Supplemental Figure 9, D–F). We found that the mean methylation level of CpGs within the *TNF* promoter was significantly downregulated in M1 hMDMs, but the mean methylation level of the *IL6* promoter containing CpGs sites –328 and –307 upstream of transcription starting site (TSS) had a decreased tendency. However, the methylation level of *IL1B* promoter was still unchanged compared with the control. Consistent with the above results, the mean methylation levels of *IL6* and *TNF* but not *IL1B* promoters in macrophages with RFX1 overexpression were reduced significantly as well (Figure 8, C and D, and Supplemental Figure 9, G–I). We overexpressed RFX1 with APOBEC3A silencing and examined the methylation changes of *IL6* and *TNF* promoter. The results show that overexpression of RFX1 after interfering with APOBEC3A expression in hMDMs had no significant effect on the mean methylation levels of *IL6* and *TNF* promoters (Supplemental Figure 9, J and K). These results suggest that RFX1 might promote proinflammatory macrophage polarization by inducing demethylation of *IL6* and *TNF* promoters by APOBEC3A. In addition, we also found the increased expression of APOBEC3A in CD14^+^ monocytes from patients with SLE (Supplemental Figure 10A). Therefore, we determined the methylation levels of *IL6*, *TNF*, and *IL1B* promoters in monocytes/macrophages. Compared with HCs, the methylation levels in the promoter of *TNF* in CD14^+^ monocytes from patients with SLE were significantly decreased (Figure 8E and Supplemental Figure 10B), but the mean methylation level of the *IL6* promoter had a decreased tendency in CD14^+^ monocytes from patients with SLE (Figure 8F and Supplemental Figure 10C). There still were no significant differences in the levels of the mean methylation of *IL1B* promoters between the CD14^+^ monocytes from patients with SLE and HCs (Supplemental Figure 10D). The methylation levels in the promoters of *TNF* and *IL6* in M1 hMDMs from patients with SLE were decreased significantly but not in *IL1B* promoter (Figure 8, G and H, and Supplemental Figure 10, E–G).

### ADP may be an endogenous antagonist of RFX1.

**Figure 9:**
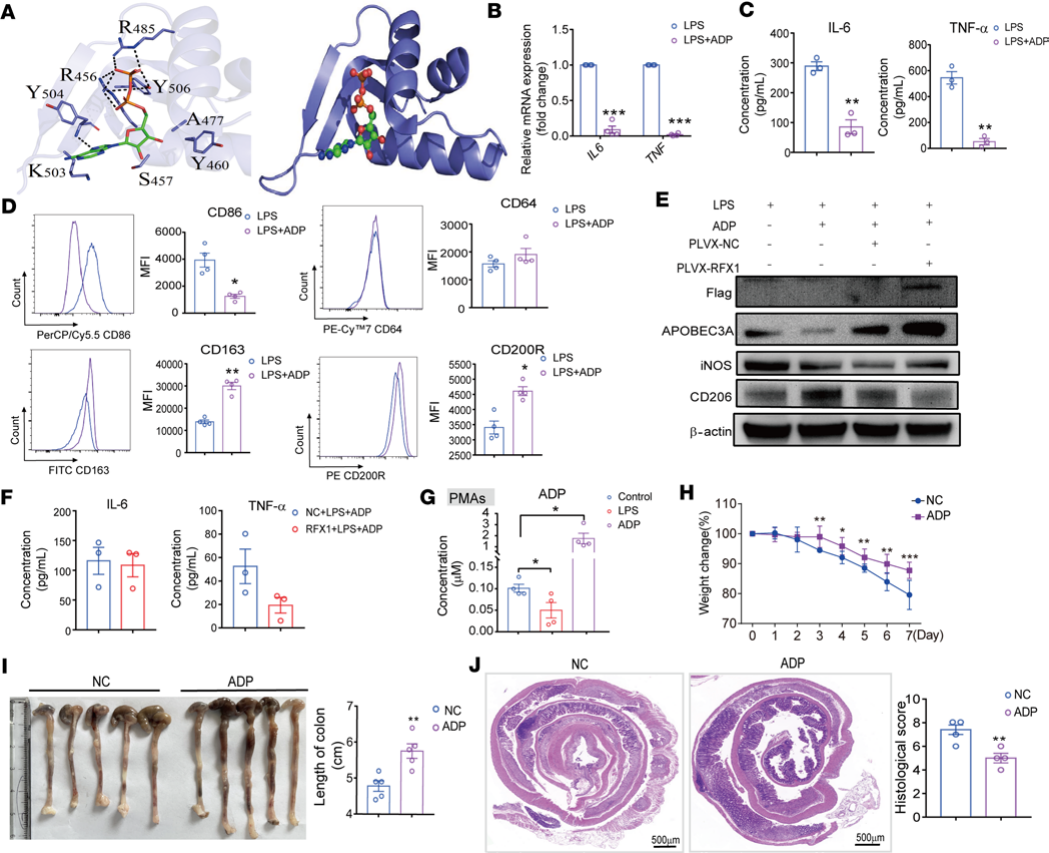
ADP inhibited the regulatory function of RFX1. (**A**) The predicted 3-dimensional interaction mode of ADP and RFX1 combination. (**B**) Relative mRNA expressions of *IL6*, *IL1B*, and *TNF* in M1 hMDMs with or without ADP treatment (1 mM) (*n* = 4). (**C**) The concentrations of IL-6 and TNF-α in M1 hMDMs culture supernatant with or without ADP treatment (*n* = 3). (**D**) Representative flow cytometry histograms and MFI statistics of CD86, CD64, CD163, and CD200R in each group (*n* = 4). (**E**) Western blot was used to detect the protein expression in hMDMs with indicated treatment. (**F**) The concentrations of IL-6 and TNF-α in culture supernatant from pLV-NC and pLV-RFX1-infected M1 hMDMs treated with ADP (*n* = 3). (**G**) The concentrations of ADP in PMAs with indicated treatment (*n* = 4). (**H**) Daily weight change of mice with colitis with or without ADP administration (*n* = 5 per group). (**I**) Representative images of colon and measured colon length (*n* = 5 per group). (**J**) Representative images of H&E staining and the histological score of the colon from mice with colitis (*n* = 4 per group). Scale bar: 500 mm. Data represent mean ± SEM, using 2-tailed Student’s *t* test. *P < 0.05, **P < 0.01, ***P < 0.001.

RFX1 is identified as a DNA binding protein that targets particular X-box sequences in MHCII genes. It has an evolutionarily conserved DNA binding domain (DBD), presenting at the core of the RFX1 protein, and binds to the X-box motif present in targeted DNA (28). Here, the molecular docking technology was used to explore the potential small molecule inhibitors for the DNA binding region of the RFX1 protein. As shown in the 3D diagrams of ADP docking with RFX1 in Figure 9A, ADP was found to likely bind to the DBD of RFX1 and may influence the function of RFX1. To test this prediction, we treated M1 hMDMs with ADP under different concentrations and found that the expression of target gene *APOBEC3A* but not *RFX1* was significantly decreased in hMDMs with ADP treatment (1 mM) compared with NC (Supplemental Figure 11, A and B). In addition, ADP treatment also decreased the LPS-stimulated APOBEC3A protein expression in hMDMs (Supplemental Figure 11C). Meanwhile, the mRNA and protein expression levels of IL-6 and TNF-α were also significantly reduced in M1 hMDMs after ADP treatment (Figure 9, B and C). Moreover, we found that the expression of M1-related molecule CD86 but not CD64 was downregulated, while the expression levels of M2-related molecules CD163 and CD200R were significantly increased in ADP-stimulated M1 hMDMs (Figure 9D). Overexpressing RFX1 could further counteract the inhibition of APOBEC3A expression and promotion of CD206 expression induced by ADP treatment (Figure 9E). In addition, ADP treatment counteracted the increased expression levels of IL-6 and TNF-α in hMDMs caused by RFX1 expression (Figure 9F). We further determined the effect of ADP treatment on PMAs and found that ADP reduced the mRNA expression of *Apobec3* but not *Rfx1* at a 1 mM concentration (Supplemental Figure 11, D and E). As determined by Western blot, the protein levels of RFX1 and Apobec3 were decreased after ADP treatment (Supplemental Figure 11F). ADP treatment decreased the mRNA expression of TNF-α and IL-1β and inhibited the protein expression of IL-6 and IL-1β in PMAs (Supplemental Figure 11, G and H). ADP treatment significantly increased the mRNA and protein expression of M2-related IL-10 (Supplemental Figure 11, I and J). We also found that ADP treatment increased the methylation levels of *IL6* and *TNF* promoters in LPS-induced hMDMs (Supplemental Figure 11, K and L).

Furthermore, we detected the ADP concentration in PMAs. It was found that the concentration of ADP in PMAs induced with LPS was significantly reduced compared with control, and ADP incubation can increase the intercellular ADP level (Figure 9G). Finally, we investigated the effect of ADP administration on inflammatory response in DSS-induced mice. We found that ADP administration alleviated weight loss and colon tissue shortening in mice with colitis (Figure 9, H and I). The degree of damage in colon tissue was reduced, and the tissue structure was more intact in mice with colitis with ADP administration (Figure 9J). Therefore, ADP administration may be therapeutic for autoimmune inflammation.

## Discussion

Transcription factors and epigenetic regulation play an important role in regulating macrophage polarization and autoimmune inflammation. For example, histone 3 lys27 tri-methyltransferase Ezh2 epigenetically inhibited antiinflammatory mediator Socs3 in macrophages by mediating H3K27me3, which promoted autoimmune inflammation in DSS-induced colitis and experimental autoimmune encephalomyelitis (29). Mef2c promoted M1 macrophage polarization by directly activating the transcription of *Il2a* and *Il12b*, facilitating resistance to *Listeria monocytogenes* infection and susceptibility to colitis (30). RFX1 is an evolutionarily conserved transcriptional factor that influences a wide range of cellular processes such as cell cycle, cell proliferation, differentiation, and apoptosis by regulating target genes involved in these processes. Previous studies found that RFX1, as a transcription activator of hepatitis B virus (HBV) enhancer I, could promote the transcription of HBV major surface antigen genes (28, 31, 32). RFX1 can also activate the expression of the target gene *Itga6* in Sertoli cells to promote testicular development (33). In contrast, RFX1 also represses gene transcription through its repression domain. For example, RFX1 could bind to the promoter region of *Id2* to inhibit gene expression (34). Our previous study also revealed that RFX1 repressed *IL17A* gene expression, and RFX1 deficiency in CD4^+^ T cells promoted *IL17A* gene expression and Th17 cell differentiation (20). However, the roles and mechanisms of RFX1 regulating innate immune response remain unclear. Here, we discovered that the RFX1 expression was increased in LPS-induced M1 macrophages; this effect is distinct from its expression in Th17 cells (20). RFX1 promoted M1 macrophage polarization characterized by increasing the release of proinflammatory cytokines in vitro. The ChIP-Seq technology was used to identify potential target genes directly regulated by RFX1. We finally found that RFX1 could promote APOBEC3A expression by directly binding to the enhancer region of *APOBEC3A*.

The different roles of RFX1 in T cells and macrophages revealed the complexity of RFX1 as a therapeutic target for SLE. Our results have found that ADP can inhibit the function of RFX1. Therefore, to evaluate the effect of systemic inhibition of RFX1 on autoimmunity, the administration of ADP in an SLE mouse model will be used to evaluate the effects of RFX1 inhibition on macrophage polarization and Th17 differentiation in vivo as well as the ultimate impact on the development of SLE. In addition, macrophage-targeted drug delivery systems such as macrophage-derived extracellular vesicles had been applied to the treatment of autoimmune diseases. We believe that, with the development and maturity of targeted drug delivery systems, targeting different types of immune cells for the expression and activity of RFX1 is an important research direction in the treatment of SLE.

The human genome codes for 7 paralogs of *APOBEC3* (*APOBEC3A* through *APOBEC3H*) containing zinc-binding motifs and have cytosine deamination activity, and only *APOBEC3* appears to have a protective immunity function among the *APOBECs* (*APOBEC1* through *APOBEC4*) (35). *APOBEC3A* belongs to the *APOBEC3* family, which catalyzes the conversion of cytosine on single-stranded DNA/RNA into uracil in cells and is an important antiviral function factor in the innate immune system (36, 37). *APOBEC3A* had been identified to limit exogenous retroviruses and participated in the elimination of exogenous and endogenous DNA in hMDMs, and it had anti-DNA and RNA virus activity, which was essential for host defense (38–40). *APOBEC3A* in turn generated new autoantigens, and it initiated and even amplified autoimmune responses by enhancing RNA editing (41). These studies all suggest that *APOBEC3A* might play an important role in the pathogenesis of autoimmune diseases. Recent studies revealed that APOBEC3A also was involved in promoting the expression of inflammatory cytokines in M1 macrophages by mediating RNA editing of polarization-related genes. *APOBEC3A* KO inhibited LPS and IFN-γ–induced human M1 polarization and promoted M2 polarization characterized by decreased expression of proinflammatory genes *IL6*, *IL23A*, and *IL12B* and secretion of cytokines TNF-α, IL-1β, and IL-6. Meanwhile, CD86 expression was inhibited and glycolysis was promoted (25). According to our results, APOBEC3A/Apobec3 overexpression can induce proinflammatory cytokines and promote M1 macrophage polarization in PMAs and hMDMs; these effects suggest that RFX1 induces APOBEC3A/Apobec3 expression to promote the inflammatory response.

Currently, the most widely accepted DNA demethylation involves the oxidation of mC and production of 5-hydroxymethylcytosine (hmC), 5-formylcytosine, and 5-carboxylcytosine (caC) via stepwise oxidation (42, 43). The latter 2 oxidized mC bases can be removed by thymine DNA glycosylase (TDG) and resultant repair for DNA demethylation (44). Pathways invoking mC deamination, hmC deamination, or caC deamination have all been proposed as possible contributors to DNA demethylation. Wijesinghe et al. found that APOBEC3A could deaminate mC efficiently, and this activity was comparable with its cytosine-to-uracil (C-to-U) deamination activity in an in vitro deamination assay (27). Prior biochemical work also shows that Cdar1 and Apobec3 discriminate against this hmC modification (45, 46). Therefore, we hypothesized that APOBEC3A might regulate proinflammatory M1 gene expression by inducing DNA demethylation during M1 macrophage polarization. As expected, APOBEC3A overexpression reduced the methylation levels in *IL6* and *TNF* promoter regions and significantly increased the expression of these proinflammatory cytokines. Accumulating evidence has also uncovered that the abnormal DNA hypomethylation in autoimmune-related genes, including CD11a (*ITGAL*), perforin (*PRF1*), CD70 (*TNFSF7*), and CD40 ligand (*TNFSF5*) in T cells is a crucial hallmark in SLE (47). Increased DNA demethylation in CD4^+^ T cells promoted self-reactive T cell activation and contributed to the pathogenesis of SLE (48, 49). Consistent with previous research, we also found that the methylation levels of *IL6* and *TNF* promoters were decreased and that RFX1 and APOBEC3A expression levels were increased in monocytes and macrophages from patients with SLE. These results suggest that the hypomethylation regulated by the RFX1/APOBEC3A pathway might contribute to macrophage-induced inflammation in SLE pathogenesis.

Macrophage phenotypes depend on mitochondrial function and ATP/ADP homeostasis. It had been found that M1 macrophages had the lowest but M2 macrophages had the highest level of ATP (50). In addition, the metabolic profiling detected by 1D ^1^H NMR-based metabolomics in macrophages indicated increased oxidative stress, decreased mitochondrial respiration, increased intracellular ATP in M1 macrophages, and increased intracellular ADP and adenosine monophosphate (AMP) in M2 macrophages (51). ADP plays an important role in vascular and cellular response and is immediately released to promote platelet aggregation during inflammation and injury (52). The M2 polarization of alveolar macrophages correlated with asthma severity in humans, and impaired M2 polarization exhibited less eosinophil recruitment and lung inflammation (53). The extracellular ADP was enriched in bronchoalveolar lavage fluid of asthmatic patients, and it could aggravate airway inflammation and induce mast cell infiltration in the ovalbumin-induced asthma model (54). To further investigate the effect of ADP on the inflammatory response, we detected the effect of ADP on macrophage polarization and found that ADP significantly decreased the expression of M1-related proinflammatory cytokines such as IL-6 and TNF-α but increased the expression of M2-related proteins such as CD163 and CD206 in hMDMs. However, ADP treatment neutralized the protein expression of IL-6 and TNF-α in hMDMs promoted by RFX1 overexpression. Moreover, the mRNA and protein expression levels of proinflammatory cytokines were also decreased in PMAs treated with ADP compared with the NC group. We gave continuous administration of ADP to mice with DSS-induced colitis and found that ADP could inhibit the morbidity of mice with colitis and reduce intestinal damage. The above research hinted that ADP might serve as an RFX1 inhibitor to restrain M1 macrophage polarization.

LPS has been considered a risk factor for SLE (55, 56). Recent studies have shown that the LPS level in serum from patients with SLE was elevated, and intestinal LPS biosynthesis in patients with SLE and mouse models were significantly enhanced, which induced proinflammatory activity (57, 58). Our study also found that LPS treatment reduced the concentration of ADP in PMAs in vitro. Therefore, we hypothesized that abnormally elevated LPS in patients with inflammatory and autoimmune diseases reduced the concentration of ADP in macrophages, thereby activating the regulatory function of RFX1 and promoting the inflammatory response. In addition, it is also possible that the imbalance of energy metabolism of macrophages in patients with SLE and IBD leads to the abnormal decrease of ADP levels, which exacerbates the development of these diseases. It’s worth noting, however, the impact of the cellular receptor of ADP and its related metabolites on the inflammatory response. A previous study reported that ADP caused a significant change in gene expression patterns and upregulation of several genes associated with inflammation and atherogenesis by the PY receptor (59). Therefore, our current study does not exclude the effect of exogenous ADP through its cellular receptor during macrophage polarization.

In summary, our study found that RFX1 overexpression induced M1 macrophage polarization and promoted autoimmune inflammation. Rfx1 deficiency in macrophages alleviated intestinal inflammation in mice with colitis and kidney damage in lupus-like mice. Mechanistically, RFX1 activated *APOBEC3A* transcription, which caused DNA demethylation of *IL6* and *TNF* promoters. We identified that ADP might function as an inhibitor of RFX1 to inhibit the proinflammatory response. Our study determined the role of the RFX1/APOBEC3A pathway in macrophage proinflammatory polarization and considers a potential therapeutic small molecule-ADP to treat autoimmune inflammation.

## Methods

Supplemental Methods are available online with this article.

### Mice.

Rfx1^fl/fl^ (WT) mice were constructed by Shanghai Model Organisms Center (20), and Lyz2-Cre mice were purchased from Shanghai Model Organisms Center Inc. Rfx1^fl/fl^ was backcrossed for 8 generations to the Lyz2-Cre mice to generate CKO mice. Mice with matched sex were used for experiments at 8–10 weeks of age. The matched-sex C57BL/6 mice (8 weeks) were bought from Hunan Sja Laboratory Animal Co. All mice were housed under a 12-hour light/dark cycle at 20°C–25°C and 35%–75% humidity in specific pathogen–free condition and fed with sterile pellet food and water.

### Cell culture and transfection.

The formation of PMAs was induced by 4% thioglycollate medium brewer modified (BD Biosciences, catalog 7009838). After i.p. perfusion, macrophages were completely cultured with DMEM containing 10% FBS (Thermo Fisher Scientific). The human CD14^+^ monocytes were separated from PBMC by density gradient centrifugation (320*g*, 20°C, and centrifugation for 30 minutes) and separated by CD14 MicroBeads (Miltenyi Biotec, catalog 130-050-201). The CD14^+^ monocytes were induced by M-CSF (Absin, catalog abs04696) (50 ng/mL) for 8 days to differentiate into hMDMs. Blood was obtained from age and sex-matched HCs or patients with clinically indicated SLE by the classification criteria for SLE (1997/2012) at the Second Xiangya Medical School, Central South University (60). Macrophages were stimulated with LPS (1 μg/mL) (MilliporeSigma, catalog L4391) or IL-4 (20 ng/mL) (PeproTech, catalog 200-04 or 214-14) for 24 hours to promote macrophage polarization. The IFN-γ (20 ng/mL) was used to treat hMDMs (PeproTech, catalog 300-02) or PMAs (PeproTech, catalog 315-05). Overexpressing RFX1/Rfx1, shRfx1, and shAPOBEC3A in pLV-Flag lentiviral vector and NC (pLV-NC) were purchased from Shanghai Genechem Co. The 293T (CRL-3216) and B16F10 cells (CRL-6475) were purchased from ATCC. Overexpressing APOBEC3A/Apobec3 in pLV-Flag lentiviral vector (pLV-APOBEC3A) and NC (pLV-NC) were purchased from Ubigene Biosciences Co.

### Macrophage function assay.

The phagocytosis assay of macrophages engulfing pHrodo-labeled bacteria was performed by pHrodo Green *E. coli* BioParticles conjugate for phagocytosis (Thermo Fisher Scientific, catalog P35366) according to instructions. In brief, pHrodo Green *E. coli* BioParticles conjugate was added to the cell culture supernatant, which is engulfed by macrophages. Flow cytometry was used to detect the fluorescence level of macrophage. The experimental procedure for detecting the ability of macrophages to regulate T cell differentiation is as follows: the CD4^+^ T cells in spleen from the C57BL/6 mice were sorted by CD4 magnetic beads (Miltenyi Biotec, catalog 130-117-043) and stimulated with 2.5 μg/mL anti-CD3 (eBioscience, catalog 16-0031-82) and 3 μg/mL anti-CD28 (eBioscience, catalog 16-0281-82) for 48 hours; the activated CD4^+^ cells were coincubated with different macrophage culture supernatants, and the differentiation ratio of CD4^+^ T cells was measured 24 hours later.

### DSS-induced colitis.

DSS (MP Biomedicals, catalog MFCD00081551) was added to the daily drinking water of mice, with a final concentration of 3%. The remaining water volume was checked every day and supplemented in time. In general, mice were killed 7 days later or killed on the ninth day after the withdrawal of drugs for 2 days. The changes in the body weight of mice were recorded every day. The weights of mice on each day were normalized to day 0.

### IMQ-induced lupus-like mice.

A total of 1.25 mg of 5% IMQ cream (MED-SHINE, China) was administered through the topical application on the right ear of IMQ-treated mice 3 times weekly for up to 10 weeks (24).

### Histological analysis and score.

Colon and kidney tissues were paraffin embedded and then cut into 5 mm sections. Sections were stained with H&E. The histological score of colon tissue was evaluated by tissue damage and innate inflammatory cell infiltration. The scoring criteria was as follows: 0, absent tissue damage or inflammatory infiltration; 1, focal epithelial damage in the colon (<5%) or increased infiltration in crypt basis; 2, epithelial damage (5%–20%) or a cluster infiltration of inflammatory cells in submucosa; 3, extensive epithelial damage in the colon (20%–50%) or large infiltration in muscularis mucosae; and 4, extensive infiltration in the submucosa or extensive epithelial damage in the colon (>50%). The scoring criteria for the kidney indicate that renal histopathological changes were quantitated with the method described before (61). Pathological changes in the kidney were assessed by evaluating glomerular activity (e.g., glomerular proliferation, karyorrhexis/fibrinoid necrosis, cellular crescents, hyaline deposits, and inflammatory cells) and the tubulointerstitial activity (e.g., interstitial inflammation, tubular cell necrosis, and flattening and tubular distension). Sections were scored using a 0–3 scale for glomerular activity, as follows: 0, no lesions; 1, lesions in < 25% of glomeruli; 2, lesions in 25%–50% of glomeruli; and 3, lesions in > 50% of glomeruli. Tubulointerstitial activity was scored using a 0–4 scale, as follows: 0, no lesions; 1, lesions in 1%–10%; 2, 11%–25%; 3, 26%–50%; and 4, 51%–100%. The scores for individual pathological features were summed.

### Single-cell RNA-Seq data retrieval and analysis.

To identify the RFX1 expression in macrophages from HC or patients with SLE, we retrieved single-cell RNA-Seq data sets (GSE179633) from the National Center for Biotechnology Information Gene Expression Omnibus (GEO) database (https://www.ncbi.nlm.nih.gov/geo/) and performed standard procedures (62). The function of Average Expression of Seurat (V4.0.3) was applied to calculate the average gene expression of each cluster or cell type. The distribution of single cells in clusters, sample groups, or each sample was visualized by t-distributed Stochastic Neighbor Embedding (t-SNE) plots. Some specific gene expression levels were plotted by feature plots and stacked violin plots. Other R packages, such as pheatmap, ggplot2, dplyr, and RColorBrewer, were used for visualization. We followed the same steps as above for the subclustering analysis.

### ADP detection and in vivo administration.

The concentrations of ADP (Selleckchem, catalog S9368) in macrophages were measured by ADP Assay Kit (MilliporeSigma, catalog MAK133) following the instructions. The process is outlined as follows. A total of 1 × 10^4^ PMAs were directly cultured in the assay microplate and stimulated by LPS or ADP for 24 hours. Working agents were used to lysis cells to release ATP and ADP. In the presence of fluorescein, ATP immediately reacts with the substrate (D-fluorescein) to produce luminescence. ADP was converted to ATP by an enzyme reaction, and this newly formed ATP then reacted with D-luciferin in the previous step. The luminescence (relative light units) was read by a luminometer immediately, using the value obtained from the appropriate standards to plot a standard curve and determine the amount of ADP present in the samples. Mice with colitis in the NC and the ADP groups were all constructed by 3% DSS. The ADP (250 mg/kg) was i.p. injected into the ADP group, while mice from the control group were i.p. injected with the same amount of saline every day for 7 days.

### Molecular docking.

Small molecules are docked into the DBD of RFX1 for scoring its complementary values at the binding sites by molecular docking (63). The structures of all compounds were derived from the PubChem database (https://pubchem.ncbi.nlm.nih.gov). The crystal structure of the RFX1 was obtained from PDB (https://www.rcsb.org), and PDB no. 1DP7 was selected. The protein structures were then pretreated using Protein Preparation Wizard in the Schrodinger software package; the protonation state of amino acid residues was adjusted according to the setting pH conditions, hydrogen atoms and protein structures were completed, and the protein structure using oPLS-3E was optimized. For screening the product library, the LigPrep module in the Schrodinger software package was used to prepare these structures and generate 3D conformation. The molecular docking operation is carried out by the Glide module in the Schrodinger software package to investigate the action mode of existing inhibitors and realize the virtual screening of existing drug molecules based on molecular docking. Interaction Visualizer in the Glide module of the Schrodinger software package then was used to analyze the interaction between target proteins and compound molecules. In the receptor-ligand interaction analysis, we mainly analyzed the docking score and the corresponding binding mode between proteins and small molecules. The docking score was primarily considered. Analyzing receptor-ligand binding modes, the nonbonding interactions between proteins and different ligands were mainly analyzed, including hydrophobic interactions, hydrogen bonding interactions, salt bridging interactions, and electrostatic interactions.

### Statistics.

GraphPad Prism 7.0 was used to perform statistical analysis. Each experiment was reproduced at least 3 times. Data obtained from this study were presented as the mean ± SEM. Statistical analyses were performed by 2-tailed Student’s *t* test or 1-way ANOVA with Dunnett’s multiple-comparison test. *P* values less than 0.05 was statistically significant.

### Study approval.

All human studies were approved by the Ethics Committee of the Second Xiangya Hospital of Central South University. All participants provided written informed consent. All animal procedures were approved by the IACUC of the Laboratory Animal Research Center at the Second Xiangya Medical School, Central South University.

### Data availability.

Data for RNA-Seq and ChIP-Seq are available in the GEO database with accession nos. GSE210667 and GSE210668. To review GEO accession GSE210667, go to https://www.ncbi.nlm.nih.gov/geo/query/acc.cgi?acc=GSE210667 and enter token cbylcyoablwtzaz into the box. To review GEO accession GSE210668, go to https://www.ncbi.nlm.nih.gov/geo/query/acc.cgi?acc=GSE210668 and enter token mnmxqwokxlelfar into the box. Data are available in the Supporting Data Values file.

## Author contributions

SY and PD performed study concept and design. HC and WH performed the experiment of bisulfite sequencing PCR (BSP). QL, SJ, and XG provided technical and material support for the studies. M Zheng and ZH analyzed the data of single-cell RNA-Seq and visualized the results. M Zhao conceived the studies, interpreted the data, directed the studies, and revised the manuscript. SY and PD share the first authorship, and the order in which they are listed was determined by their workload. All authors read and approved the final paper.

## Supplementary Material

Supplemental data

Supporting data values

## Version 1 09/21/2023

In-Press Preview

## Version 2 10/23/2023

Electronic publication
