# Supplementary material for: Regulatory factor X1 induces macrophage M1 polarization by promoting DNA demethylation in autoimmune inflammation
Source: JCI Insight. 2023 Oct 23;8(20):e165546. doi: 10.1172/jci.insight.165546 (PMC10619507; doi:10.1172/jci.insight.165546)
Supplement: Supplemental data [file jciinsight-8-165546-s220.pdf]

Supplemental Methods

Immunofluorescence staining (IF)

The hMDMs were inoculated with cover glass for growth cover. Subsequently, cells were induced with LPS (1μg/ml) or IL-4 (20ng/ml). The cells were immobilized and permeabilized with 4% paraformaldehyde containing 0.5% Triton X-100 (Sigma-Aldrich, Catalog#9002-93-1) for 30 min at room temperature after being rinsed with PBS thrice. Next, cells were blocked by 5% BSA for 20 min and hatched with anti-RFX1 antibody (Santa Cruz, Catalog#sc-374270) overnight. The cells were hatched with Alexa Flour 488 conjugated Goat anti-Mouse antibody (Abcam, Catalog#ab150081) for 1 hour after being rinsed with PBS thrice.

Mouse tissue was fixed in neutral general purpose tissue fixator (Servicebio, Catalog#G1101) and was dehydrated before paraffin embedding and sectioned into 5μm. The tissue sections were deparaffinized with turpentine before rehydration with gradients of alcohol. The antigen was retrieved by citric acid repair solution (Servicebio, Catalog#G1202) under high temperature and pressure. Opal™ Manual IHC Kit (PerkinElimer, Catalog#NEL810001KT). Tissue section were blocked by 5% BSA for 30 min and stained with anti-Il-6 (Abcam, Catalog#ab290735), anti-Rfx1 (NOVUS, Catalog#NBP1-52654), anti-Tnf-α (Abcam, Catalog#ab1793), anti-Il-1β (Abcam, Catalog#ab283818), anti-Arg-1(Abcam, Catalog# ab233548), anti-CD206 (Abcam, Catalog#ab64693), anti-iNOS (Abcam, Catalog#ab115819), anti-CD16/32 (RD, Catalog#AF1460), anti-C3(Abcam, Catalog#ab200999) or anti-IgG (Abcam, Catalog#ab205724) respectively. The tissue sections were then incubated with HRP

anti-Rabbit IgG antibody (Abcam, Catalog#ab288151) or Donkey Anti-Rabbit IgG H&L (HRP) (Abcam, Catalog#ab205722). The secondary antibody and Opal Fluorophore (PerkinElimer, Catalog#NEL810001KT) respectively for 30 mins at room temperature. DAPI (Servicebio, Catalog#G1012) staining was used to counterstain the nuclear, and the images were acquired by PerkinElmer multispectral Pathology Analysis System (Akoya).

Flow cytometry analysis

Colon, kidney or tumor tissues were isolated from sacrificed mice. The chopped kidneys were digested by collagenase IV (Gibco, Catalog#17104019). The colon tissues were rinsed and digested by Dispase II protease (Sigma, Catalog#CAS42613-33-2) and collagenase IV (Sigma, Catalog#CAS9001-12-1) after being cut into pieces with scissors. Tumor tissues were digested by tumor dissociation kit (Miltenyi Biotec, Catalog#130-096-730) according to the instructions of the manufacture. Cells were incubated with antibodies for 30min on ice in the dark. The PMAs and hMDMs were directly scraped by cell scrapers. All antibodies were indicated in Supplementary Table 1. Cells were fixed and permeabilized with Cytofix/Cytoperm (BD, Catalog#554714), according to the instructions described by manufacturer. The staining cells were analyzed on Northern Lights flow cytometer (Cytek) and the data were analyzed using FlowJo software.

Quantitative real-time PCR (RT-qPCR)

Total messenger was isolated by Trizol and quantified using NanoDrop 2000 (Thermo). The 1μg RNA was reversed to complementary DNA using PrimeScript^{TM} RT reagent

kit with gDNA Eraser (TaKaRa, Catalog#RR047B). Quantitative real-time PCR was performed using SYBR® *Premix Ex* Taq™ (Tli RNaseH Plus) (TaKaRa, Catalog#RR820A) on Lightcycler 96 thermocycler (Roche). Relative expression was calculated using glyceraldehyde 3-phosphate dehydrogenase (GAPDH) as an internal control. The primers' sequences are shown in Supplementary Table 2.

Enzyme linked immunosorbent assay (ELISA)

Depending on the species, cytokines concentrations in the culture medium or serum were quantified by ELISA kits with human or mouse species reactivity according to instructions of the manufacture. The mouse TNF-alpha ELISA Kit (CUSABIO, Catalog# CSB-E04741m), mouse IL-6 ELISA Kit (CUSABIO, CSB-E04639m), mouse IL-1 beta ELISA Kit(CUSABIO, CSB-E08054m), and IL-10 Mouse Uncoated ELISA Kit (Invitrogen, Catalog#88-7105-88), human IL-6 ELISA Kit (MULTISCIENCES, Catalog#EK106/2-96), human TNF-α ELISA Kit (MULTISCIENCES, Catalog#EK182-96), human IL-10 ELISA Kit (MULTISCIENCES,Catalog#EK110/2-96), human IL-1β ELISA Kit (MULTISCIENCES, Catalog#EK101B-96) were used to detect the cytokines concentrations. The mouse IgG ELISA Kit (MULTISCIENCES, Catalog#EK271-96), mouse anti-double stranded DNA antibody (IgG) ELISA Kit (CUSABIO, CSB-E11194m) and mouse anti-nuclear Antibody (IgG) ELISA Kit (CUSABIO, Catalog#CSB-E12912m) were used to detect autoantibody concentration in serum.

Western blot (WB)

Cells were lysed by RIPA lysis buffer (Beyotime, Catalog#P0013B) with proteinase

inhibitor for 2 hours at 4°C. The BCA assay kit (Thermo Scientific, Catalog#23227) was used to quantify protein concentration. Boiling proteins in SDS-loading Buffer to realize protein denaturation. Resolved proteins with SDS-PAGE were then transferred into PVDF membrane. The membrane was blocked by 5% skimmed milk and incubated with indicated primary antibodies (anti-RFX1, Genetex, Catalog#gtx108664; anti-APOBEC3A, abcam, Catalog#ab262853; anti-Apobec3, Santa Cruz, Catalog#sc390254; anti-iNOS, Proteintech, Catalog#80517-1-RR; anti-CD206, abcam, Catalog#ab64693) at 4°C overnight. The membrane was hatched with HRP-conjugated secondary antibodies at room temperature for 1 hour after washing with TBST three times. Blotting bands were detected by ECL Western Blotting Detection Reagent (BD) and quantified by Image J.

RNA-seq

The whole genome RNA was extracted using TRIZOL. RNA-seq was conducted by Genergy Biotechnology (Shanghai) Co., Ltd. The process was briefly described. A two-way sequencing mode of the Illumina HiseQ sequencing platform was used to perform high-throughput sequencing for multiple samples. The Skewer software was used for dynamically remove the joint sequence and poor-quality fragment. The FASTQC software was used to quality control and statistics on the pre-processing data. The STAR software was used to compare the preprocessed sequence with the reference genome sequence of the sequenced species. The StringTie software was used to count the original sequence of known genes, and FPKM (Fragments Per Kilobase of transcript per Million fragments mapped) was used to calculate the expression. Using DESeq2

software to screen differently expressed genes between two groups according to the experimental design. |Log2FC|≥1 and p-value≤0.05 are the screening criteria for differential genes between two groups. The data of RNA-seq were available in the GEO database with the accessible codes of GEO210667.

Data analysis tools

GO, KEGG analysis were performed using a free online platform for data analysis (https://david.ncifcrf.gov/summary.jsp). Hot map, bubble diagram and volcano plot were constructed using Oebiotech Share tools, a free online platform (https://cloud.oebiotech.cn/task/). Gene set enrichment analysis (GSEA) were performed using the clusterProfler package 4.0.5 in R 4.1.1.

Chromatin immunoprecipitation (ChIP) - qPCR and sequencing (seq)

hMDMs were infected with lentivirus carrying control or Rfx1-3×Flag plasmid and stimulated with LPS for 24 hours. ChIP assay was performed as described by EZ-ChIP™ Chromatin Immunoprecipitation Kit (Millipore, Catalog#17-371). EZ-Magna ChIP™ A/G (Millipore, Catalog#17-10086) was used for ChIP-seq. Briefly, 1% formaldehyde was used to crosslink cells for 5 mins at room temperature and glycine was added to stop the reaction. Cells were washed with PBS twice and cleaved in lysis buffer containing a protease inhibitor cocktail. The pyrolysis product was then sonication to generate DNA fragments with a size of approximately 500bp. The targeted protein was then enriched with magnetic or protein G agarose beads coated with an anti-Flag antibody. The DNA fragments regulated by RFX1 were obtained by separating the DNA fragments bound to the target protein by reversed crosslinking.

DNA treated with proteinase K, and RNase was recovered by purification Kit.

The purified DNA was used as a template for SYBR Green-based real-time qPCR or used for sequencing. The following primers were used: forward: AGACTTCGGCTTCAGTGACTATCC; reverse: CTGAATTGTTCCTGGTGAGAGC. ChIP-seq was conducted by Genergy Biotechnology (Shanghai) Co., Ltd. Before data analysis, the original sequencing data were processed to remove the low-quality and splitter sequences using Skewer software. The Fastqc software was used to perform quality control of the treated sequences. The filtered sequencing data were compared with the reference genome (GRCh37/hg19) by Bowtie to obtain alignment information of ChIP-seq. Alignment peak position and alignment intensity information were found using MACS by comparing position information of reads on the genome. We used IGV 2.12.3 software to detect the significant sequences in the peak and data visualization. The data of ChIP-seq are available in the GEO database with the accessible codes of GEO210668.

Dual-luciferase reporter assay

The genomic fragment of *APOBEC3A* containing the RFX1 binding region was cloned into the pGL3-promoter vector. The coding region of *RFX1* was cloned into pcDNA3.1 vector. The pcDNA-NC or pcDNA-RFX1 was transfected into 293T cells with pGL3-*APOBEC3A* and Renilla luciferase plasmids using Lipofectamine 2000 (Invitrogen, Catalog#11668027). The Luciferase activity was determined by the dual Luciferase Assay System (Promega, Catalog#E1910).

Bisulfite sequencing PCR (BSP)

The genomic DNA extracted from cells with a Genomic DNA Extraction kit (Accurate Biology, Catalog#AG21009) was bisulfite-treated using a Bisulfite Conversion kit (Zymo, Catalog#D5005). The MethPrimer 2.0 was used to determine whether there are CpG islands in the promoter sequence of *IL6*, *TNF* and *IL1B* and designed the outer and inner primer sequences for PCR amplification. The primers' sequences are shown in Supplementary Table 3. The quality of PCR amplified products was determined by agarose gel electrophoresis. Qualified PCR products were sent to Tsingke Biotechnology Co., Ltd. for Sanger sequencing. The methylation level of CpG site was calculated by the following equation: Meth%=C/(C+T) *100%. The mean methylation level was determined by the total methylation levels of indicated CpG sites divided by the number of sites.

Supplemental Figure 1

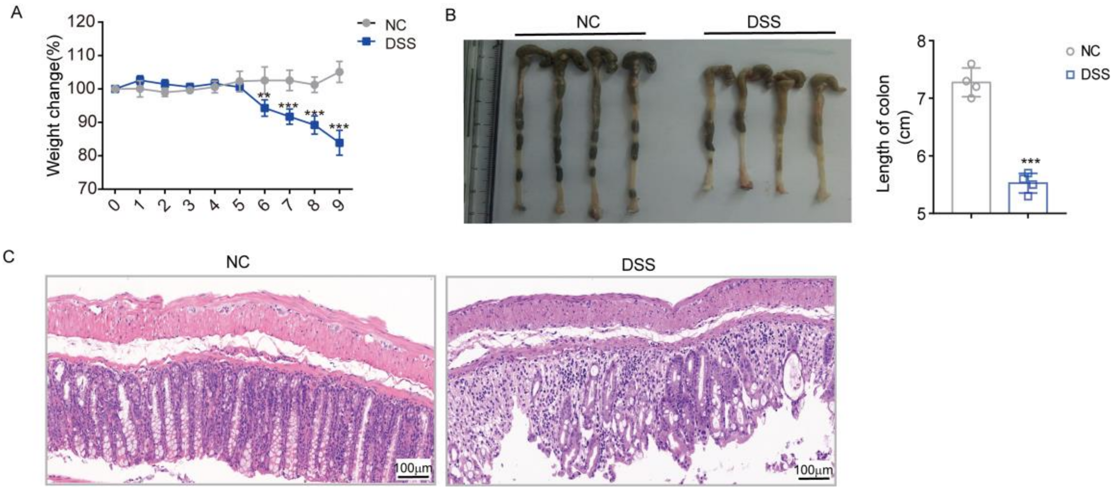

Figure 1. The DSS-induced colitis mice were constructed. A Daily weight change of mice with or without DSS treatment (n=4 per group). **B** Representative images of colon from each group and measured colon length (n=4 per group). **C** Representative image of H&E staining of the colon from control (NC) or colitis mice (DSS). Scale bar, 100μm. Data represent mean ± SEM, two-tailed Student's *t* test for **A-B**. $^{*}p<0.05$, $^{**}p<0.01$, $^{***}p<0.001$.

Supplemental Figure 2

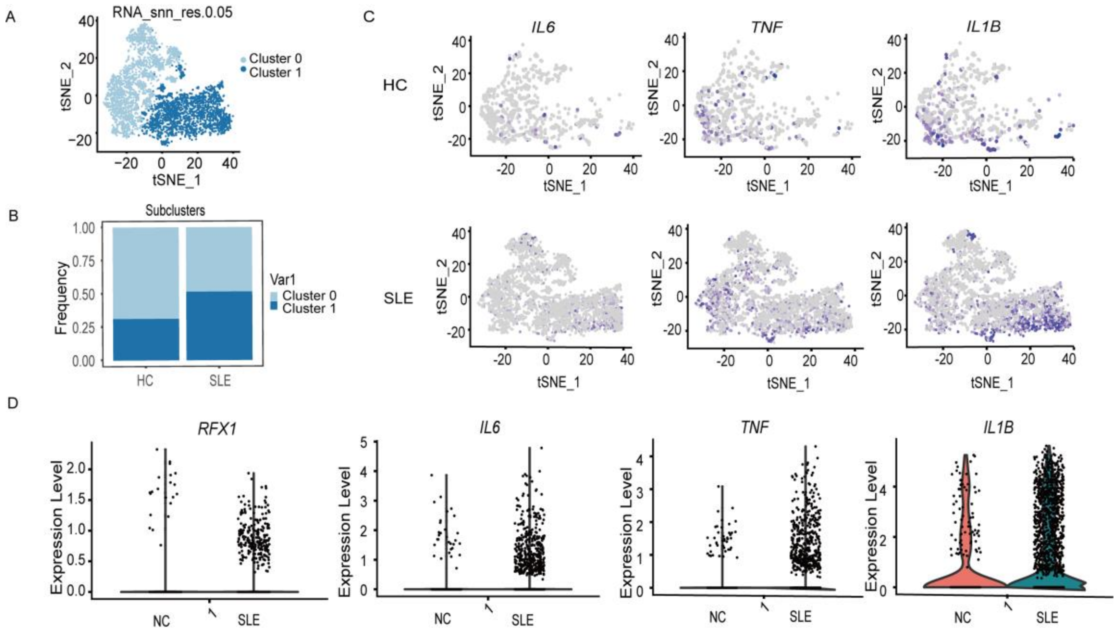

Figure 2. RFX1 expression in M1-like dermal macrophages from SLE patients was increased. A The *t*-SNE diagram shows the sub-group composition of single-cell sequencing dermal macrophages (Clusters 0 and 1). **B** The frequencies of Cluster 0 and 1 in dermal macrophage from HC or SLE patients. **C** The *t*-SNE diagram shows the expression of *IL6*, *TNF* and *IL1B* in the sub-group composition of single-cell sequencing in dermal macrophages from HC or SLE patients. **D** The expression levels of *RFX1*, *IL6*, *TNF* and *IL1B* in Cluster 1 of dermal macrophages from HC or SLE patients.

Supplemental Figure 3

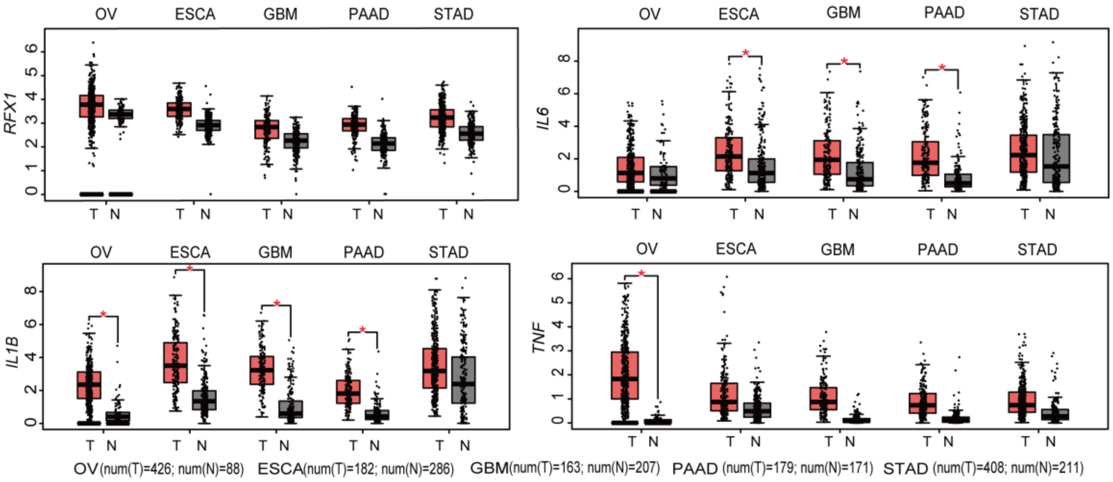

Figure 3. The expressions of RFX1 and proinflammatory cytokines were increased in multiple tumor tissue. The expression data were presented as first log2(TPM+1) transformed for differential analysis and the log2FC is defined as median (Tumor) – median (Normal). Genes with higher |log2FC| values and lower q values than 0.01 were considered differentially expressed genes. The relative expression of *RFX1*, *IL6*, *IL1B* and *TNF* in normal (N) or multiple tumor tissue (T) were showed by box plots with jitter generated by Gene Expression Profiling Interactive Analysis (GEPIA) for comparing expression in indicated cancer types. The sample size was shown in the figure.

Supplemental Figure 4

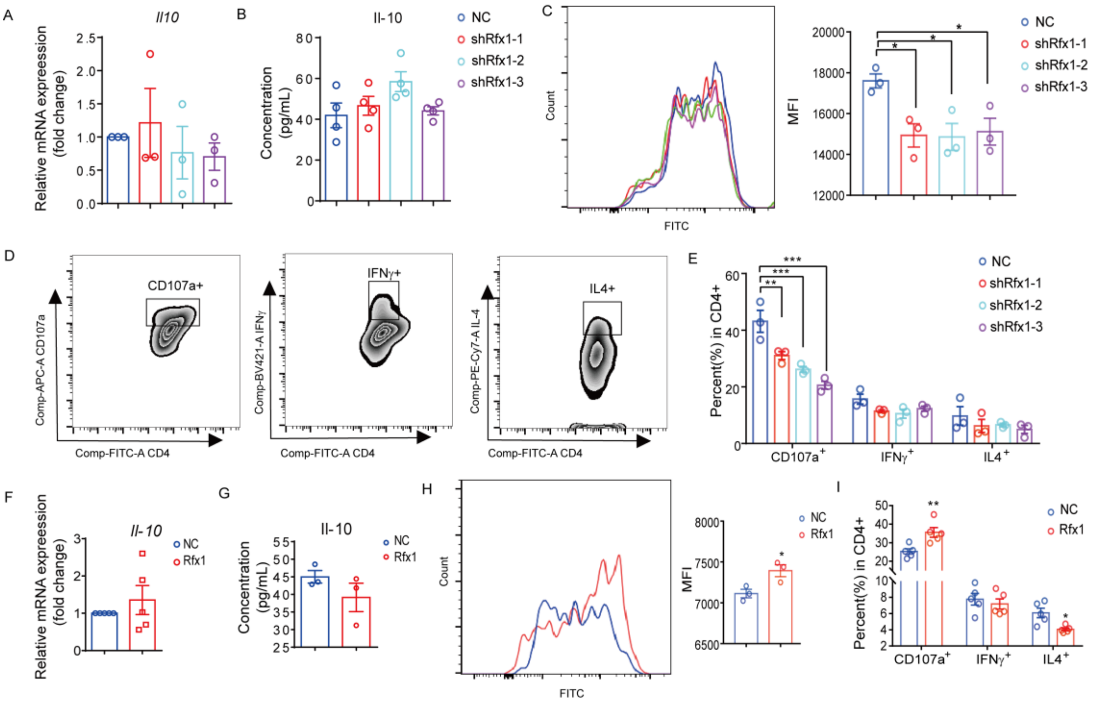

Figure 4. RFX1 regulated M1 macrophage-related functions. A-B The relative mRNA expressions (**A**) (n=3) and protein concentrations (n=4) in culture supernatant (**B**) of Il-10 were determined. **C** Representative flow cytometry histograms and mean fluorescence intensities (MFI) statistics for fluorescent particles in LPS-induced M1 PMAs transfected with pLV-NC and pLV-shRfx1 (n=3). **D** Gating strategies of $CD107a^+$, $IFN\gamma^+$ and $IL4^+$ in $CD4^+$ cells sorted from mouse spleen. **E** The proportions of $CD107a^+CD4^+$ (cytotoxic T cells), $IFN\gamma^+$ $CD4^+$ (Th1), and $IL4^+$ $CD4^+$ (Th2) in total $CD4^+$ T cells incubated different conditioned media of M1 PMAs infected with pLV-NC and pLV-shRfx1 (n=3). **F-G** The relative mRNA expressions (**F**) (n=5) and protein concentrations (**G**) (n=3) in culture supernatant of Il-10 were detected. **H** Representative flow cytometry histograms and mean fluorescence intensities (MFI) statistics for fluorescent particles in M1 PMAs (n=3). **I** The proportions of different $CD4^+$ cell subsets incubated different conditioned media of M1 PMAs (n=5). Data represent mean ± SEM, two-tailed Student's *t* test was used for **F-I**. One-way ANOVA with Dunnett's test was used for **A-E**. $^{*}p<0.05$, $^{**}p<0.01$, $^{***}p<0.001$.

Supplemental Figure 5

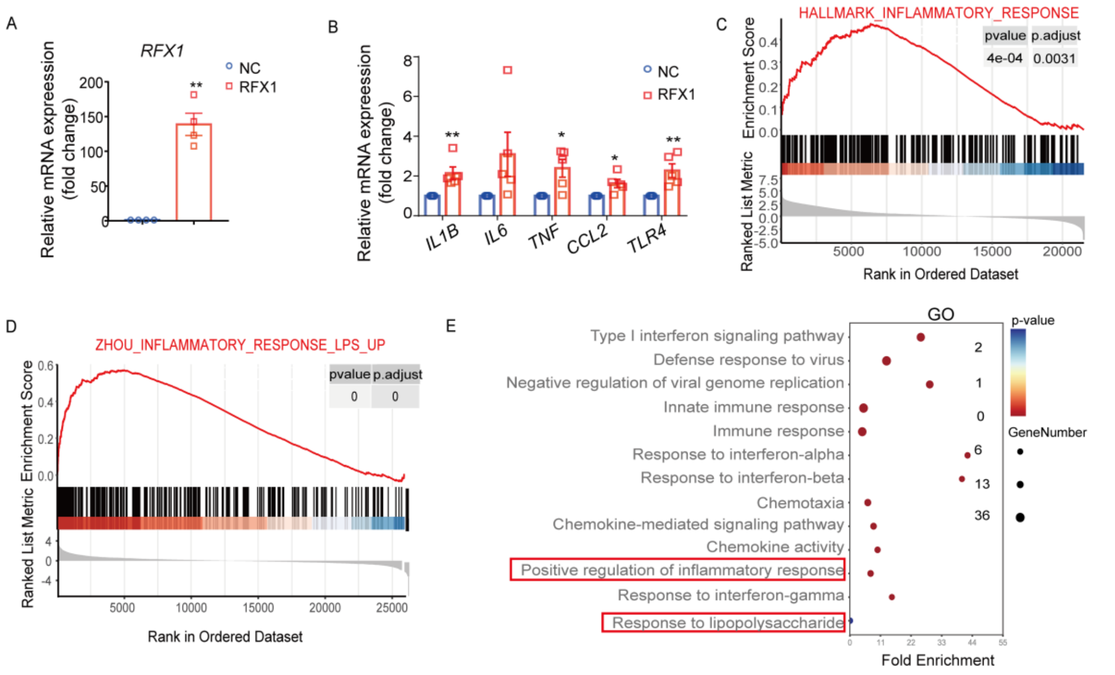

Figure 5. RFX1 overexpression promoted M1 macrophage polarization in hMDMs. A The relative mRNA expression of RFX1 in hMDMs infected with pLV-NC or pLV-RFX1 stimulated with LPS (n=4). **B** The relative mRNA expressions of indicated genes in M1 hMDMs infected with pLV-NC or pLV-RFX1 (n=5). **C-D** The GSEA pathway database was used to analyze the enrichment of differently expressed genes in representative M1-related gene sets (n=3 per group). **E** Significant enriched GO terms in up-regulated genes of M1 hMDMs infected with pLV-RFX1. Data represent mean ± SEM. Two-tailed Student's *t* test for **A-B**. $^{*}p<0.05$, $^{**}p<0.01$, $^{***}p<0.001$.

Supplemental Figure 6

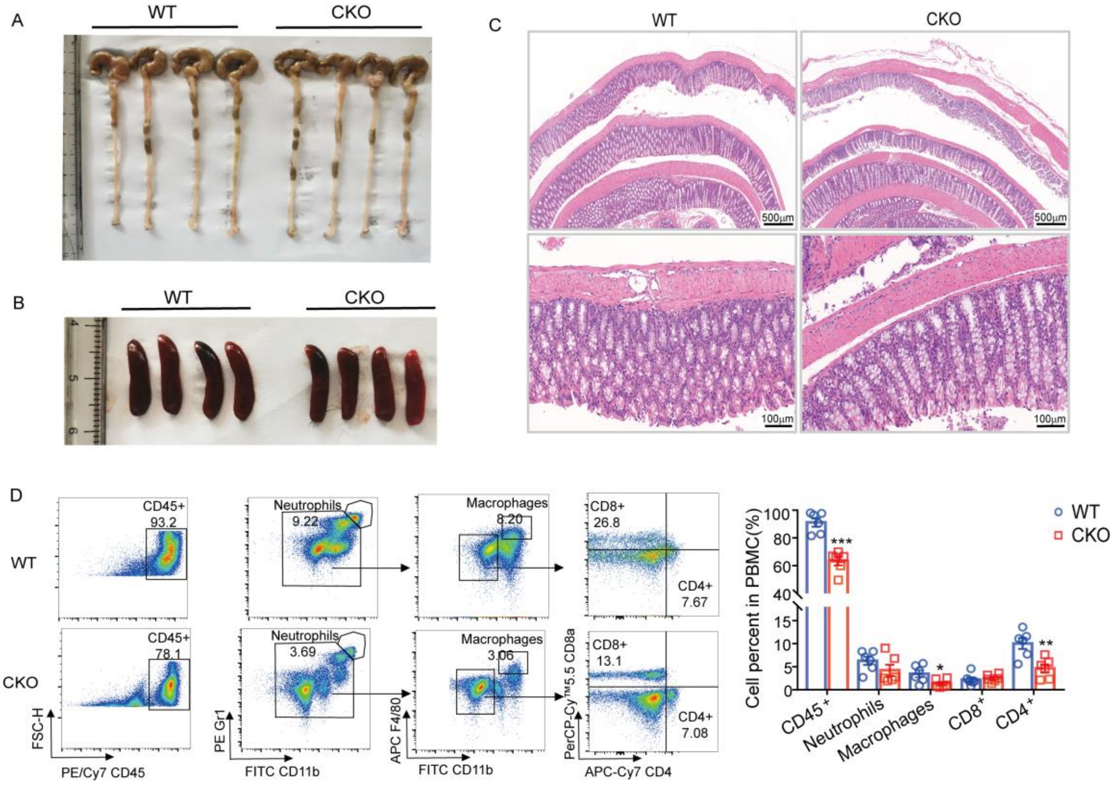

Figure 6. Rfx1 knockout in myeloid did not cause spontaneous inflammation but altered the immune cell percents in PBMCs from colitis mice. **A** The image of colon from WT and CKO mice. **B** Representative images of H&E staining of colon from WT and CKO mice. Scale bar, 500μm (above), 100μm (down). **C** The images of spleen from each group. **D** Gating strategies and proportions of $CD45^+$, $CD11b^+Gr1^+$(Neutrophils), $CD11b^+F4/80^+$(Macrophages), $CD4^+$ and $CD8^+$ cells in PBMCs from WT and CKO mice with colitis (n=6 per group). Data represent mean ± SEM. Two-tailed Student's *t* test was used. $^*p<0.05$, $^{**}p<0.01$, $^{***}p<0.001$.

Supplemental Figure 7

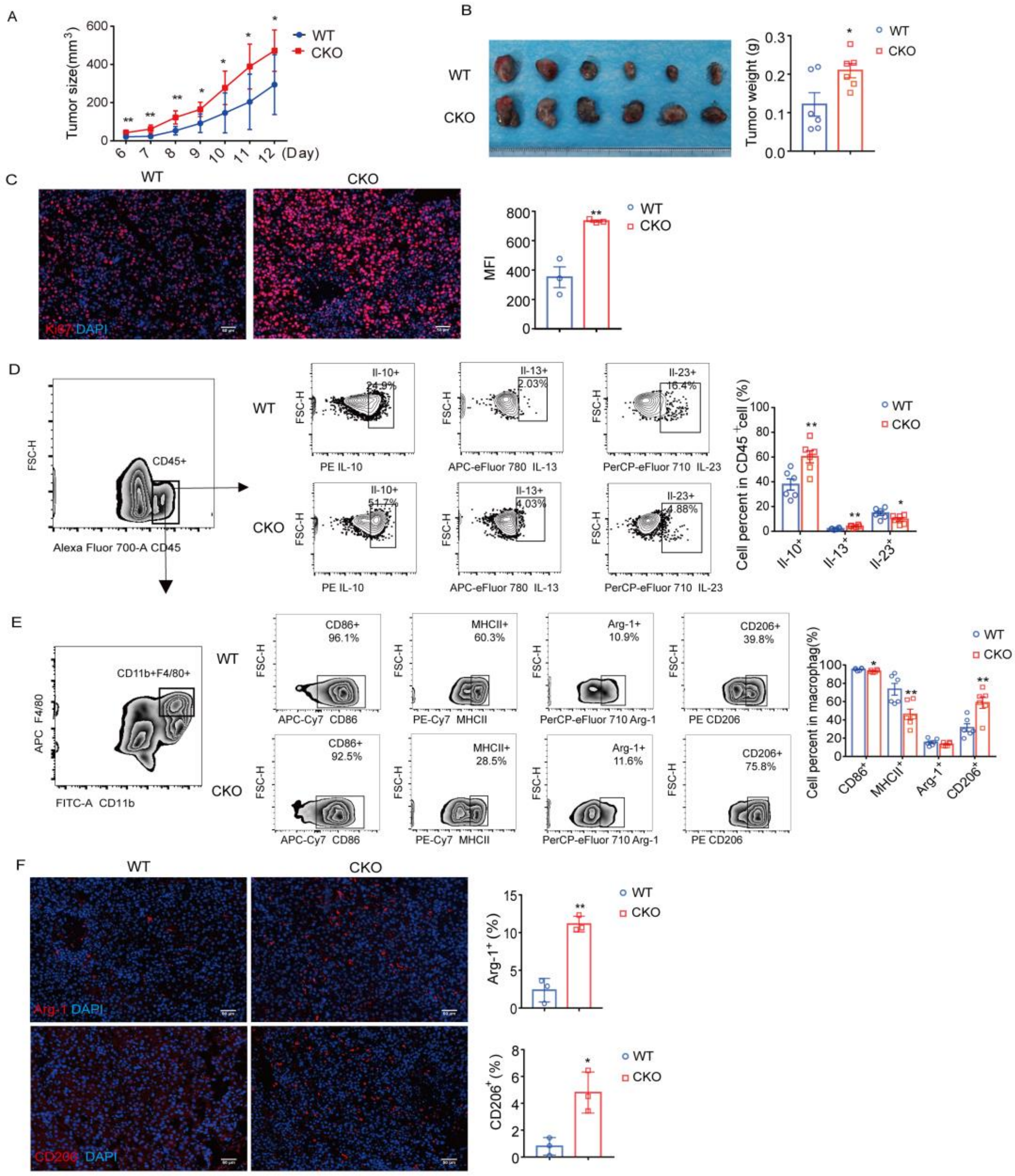

Figure 7. Rfx1 myeloid knockout promoted tumor development. A The tumor sizes of B16F10-bearing mice in indicated time (n=6 per group). **B** The image of tumor from each group and the measured weights (n=6 per group). **C** Representative images of Ki57 staining and percentages of positive cells in tumor tissue (n=3 per group). Scale bar, 50μm. **D** Gating strategies and proportions of $IL10^{+}$, $IL13^{+}$ and $IL23^{+}$ cells in immune cells ($CD45^{+}$) from tumor mononuclear cells (n=6 per group). **E** The proportions of $CD86^{+}$, $MHCII^{+}$, Arg-1^{+} or $CD206^{+}$ cells in TAMs from WT and CKO mice were displayed (n=6 per group). **F** Representative images of IF staining and percentages of Arg-1^{+} and $CD206^{+}$ cells in tumor (n=3 per group). Scale bar, 50μm. Data represent mean ± SEM. Two-tailed Student's *t* test was used. $^{*}p<0.05$, $^{**}p<0.01$, $^{***}p<0.001$.

Supplemental Figure 8

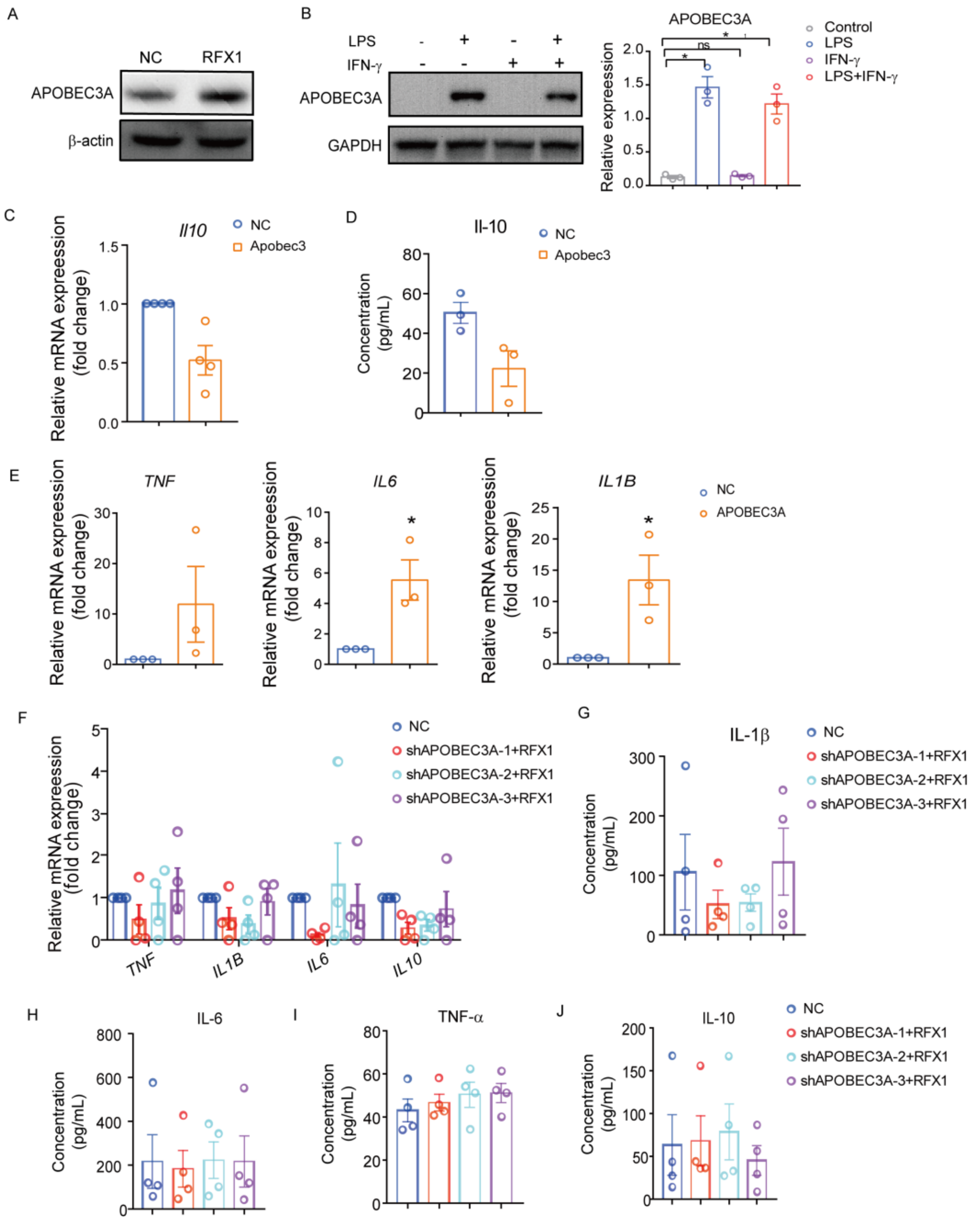

Figure 8. APOBEC3A promoted M1 macrophage polarization. A The APOBEC3A protein expression in hMDMs infected with pLV-NC and RFX1 detected by immunoblotting. β-actin was used as the loading control. **B** The APOBEC3A protein expression in hMDMs with indicated treatment (n=3 per group). GAPDH was used as the loading control. **C-D** The relative mRNA expression (**C**) (n=4) and the protein concentration (**D**) (n=3) of Il-10 in culture supernatant from PMAs with or without Apobec3 overexpression. **E** The relative mRNA expression of *TNF*, *IL6*, *IL1B* in hMDMs infected with pLV-NC or pLV-APOBEC3A (n=3). **F** The relative mRNA expression of indicated cytokine in LPS-stimulated M1 hMDMs with or without APOBCE3A knockdown and RFX1 overexpression (n=4). **G-J** The protein concentrations of IL-1β (**G**), IL6 (**H**), TNF-α (**I**) and IL-10 (**J**) in culture supernatant of in LPS-stimulated M1 hMDMs with or without APOBCE3A knockdown and RFX1 overexpression (n=4). Data represent mean ± SEM. One-way ANOVA with Dunnett's test was used for **B, F-J** and two-tailed Student's *t* test was used for **C-E**. $^{*}p$<0.05, $^{**}p$<0.01, $^{***}p$<0.001.

Supplemental Figure 9

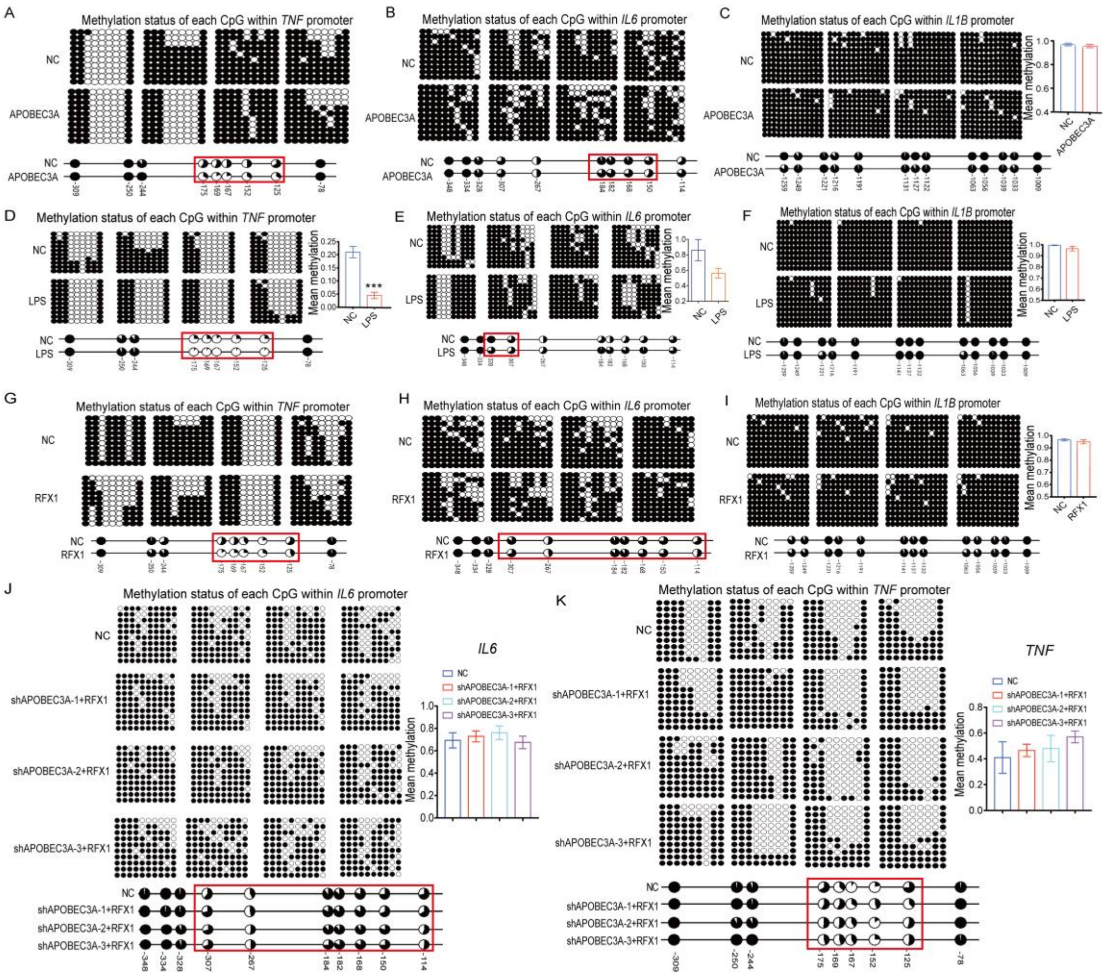

Figure 9. RFX1 regulated methylation of pro-inflammatory cytokines through APOBEC3A. A-C The methylation levels of CpG sites within *TNF* (**A**), *IL6* (**B**) and *IL1B* (**C**) promoter in hMDMs infected with pLV-NC or APOBEC3A (n=4). **D-F** The methylation levels of CpG sites within *TNF* (**D**), *IL6* (**E**) and *IL1B* (**F**) promoter in hMDMs treated with or without LPS (n=4). **G-I** The methylation levels of CpG sites within *TNF* (**G**), *IL6* (**H**) and *IL1B* (**I**) promoter in M1 hMDMs infected with pLV-NC or RFX1 (n=4). **J-K** The methylation levels of CpG sites within *IL6* (**J**) *or TNF* (**K**) promoter in LPS-stimulated M1 macrophage with or without APOBCE3A knock down and RFX1 overexpression (n=4). Ten respective clones from each group were sequenced. Data represent mean ± SEM, two-tailed Student's t test was used for **C**-**F**, **I**. One-way ANOVA with Dunnett's multiple comparison test was used in **J** and **K**. $^{*}p<0.05$, $^{**}p<0.01$, $^{***}p<0.001$.

Supplemental Figure 10

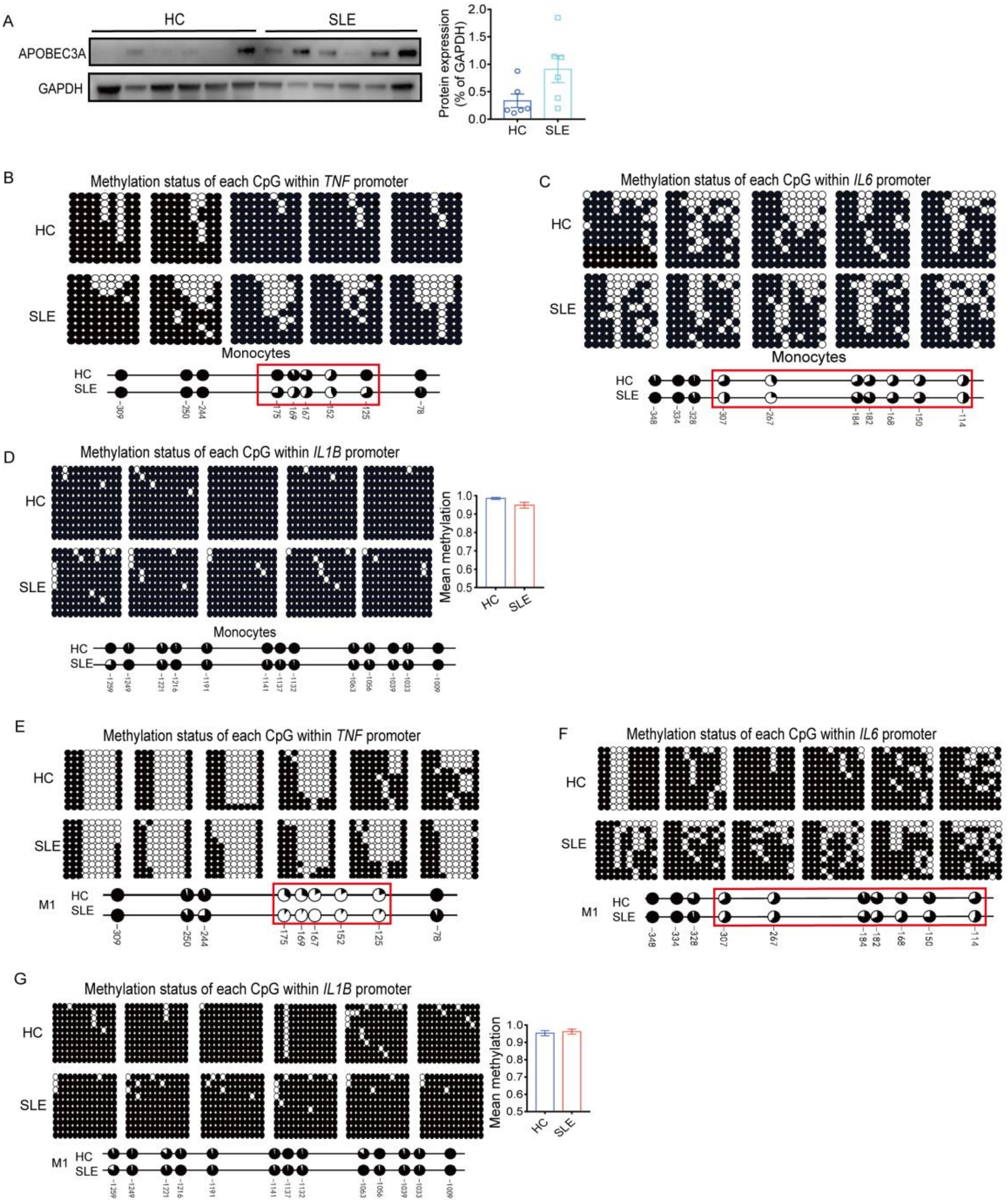

Figure 10. The APOBEC3A expression and methylation of *IL1B* promoter in monocytes/macrophages from SLE. A The APOBEC3A protein expression in monocytes from HC and SLE detected by immunoblotting (n=6 per group). GAPDH used as the loading control. **B-D** The methylation levels of CpG sites within *TNF* (**B**), *IL6* (**C**) and *IL1B* (**D**) promoter in monocytes from HC or SLE patients (n=5 per group). **E-G** The methylation levels of CpG sites within *TNF* (**E**), *IL6* (**F**) and *IL1B* (**G**) promoter in LPS-stimulated M1 macrophages from HC or SLE patients (n=6 per group). Ten respective clones from each group were sequenced. Data represent mean ± SEM, two-tailed Student's *t* test was used for **A**, **D**, **G**. $^{*}p<0.05$, $^{**}p<0.01$, $^{***}p<0.001$.

Supplemental Figure 11

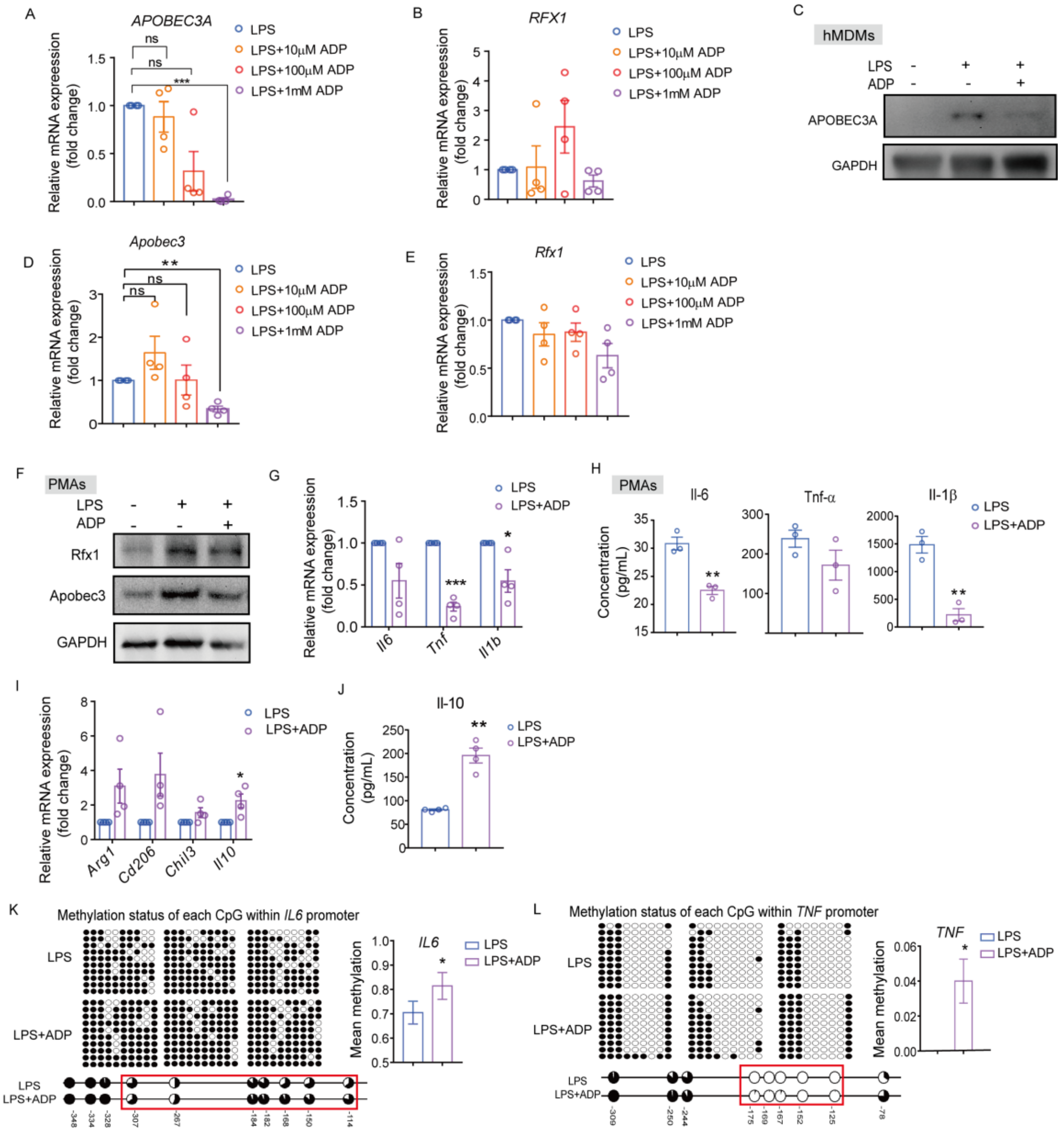

Figure 11. ADP treatment inhibited APOBEC3A expression and M1 macrophage polarization. A-B Relative mRNA expression of *APOBEC3A*(**A**) and *RFX1*(**B**) in M1 hMDMs treated by ADP with different concentrations (n=4 per group). **C** Western blot was used to detect the protein expression of APOBEC3A in hMDMs with indicated treatment. **D-E** Relative mRNA expressions of *Apobec3* (**D**) and *Rfx1* (**E**) in PMAs treated by ADP in different concentrations (n=4 per group). **F** The protein expressions of RFX1 and Apobec3 in PMAs with indicated treatment. **G** Relative mRNA expressions of *Il6*, *Tnf* and *Il1b* in M1 PMAs with or without ADP treatment (n=4 per group). **H** The concentrations of Il-6, Tnf-α and Il-1β in M1 PMAs culture supernatant with or without ADP treatment (n=3 per group). **I** Relative mRNA expressions of *Arg1*, *Mrc1* and *Chil3* in M1 PMAs with or without ADP treatment (n=4). **J** The concentration of Il-10 in M1 PMAs culture supernatant with or without ADP treatment (n=4 per group). **K-L** The methylation levels of CpG sites within *IL6* (**K**) and *TNF* (**L**) promoter in LPS-stimulated M1 macrophages with or without ADP stimulation (n=3). Data represent mean ± SEM. The *p* values were shown and assessed by one-way ANOVA with Dunnett's multiple comparison test for **A**, **B**, **D**, **E**. The two-tailed Student's *t* test was used for **G-L**. $^{*}p<0.05$, $^{**}p<0.01$, $^{***}p<0.001$.

Table 1. Antibodies for flow cytometry

Reactivity	Marker	Fluorescence	Catalog#	Brand
Human	CD200R	PE	12-9201-42	eBioscience
Human	CD163	FITC	563697	BD
Human	CD86	PerCP/Cyanine5.5	561129	BD
Human	CD64	PE-Cy™7	561191	BD
Mouse	CD45	PE/Cyanine7	103113	Biolegend
Mouse	Gr-1	PE	561084	BD
Mouse	MHCII	PE/Cyanine7	107629	Biolegend
Mouse	CD86	APC/Cyanine7	105029	Biolegend
Mouse	CD206	PE	141706	Biolegend
Mouse	Arg-1	PerCP-eFluor 710	46-3697-82	eBioscience
Mouse	B220	PerCP/Cyanine5.5	103236	Biolegend
Mouse	CD11b	FITC	11-0112-82	eBioscience
Mouse	F4/80	APC	123116	Biolegend
Mouse	CD4	FITC	557307	BD
Mouse	Zombie	NIR	423105	Biolegend
Mouse	CD4	APC-Cy™7	552051	BD
Mouse	CD8a	PerCP-Cy™5.5	551162	BD
Mouse	CD4	BV650	563232	BD
Mouse	F4/80	BV421	565411	BD
Mouse	CD45	BV785	103163	Biolegend
Mouse	Ly6G	BV570	127629	Biolegend
Mouse	Il-10	PE	561060	BD
Mouse	Il-13	APC-eFluor™780	47-7133-80	eBioscience
Mouse	Il-23	PerCP-eFluor™ 710	46-7023-80	eBioscience
Mouse	CD86	APC	558703	BD
Mouse	IFNγ	BV421	Biolegend	505830
Mouse	IL4	PE-Cy™7	BD	560699
Mouse	CD107a	APC	BD	560646

Table 2. Primers for real-time RT-PCR

Species	Primer	Sequence（5’-3’）
Mouse	Rfx1 Forward	GTCAGAAGCCAGCCCCAGTT
Mouse	Rfx1 Reverse	CTTACCTGCTGTGGCACCTGAATG
Mouse	Il-6 Forward	TAGTCCTTCCTACCCCAATTTCC
Mouse	Il-6 Reverse	TTGGTCCTTAGCCACTCCTTC
Mouse	Tnf Forward	CTGAACTTCGGGGTGATCGG
Mouse	Tnf Reverse	GGCTTGTCACTCGAATTTTGAGA
Mouse	Il1b Forward	AAGCCTCGTGCTGTCGGACC
Mouse	Il1b Reverse	TGAGGCCCAAGGCCACAGGT
Mouse	Chil3 Forward	TTTCTCCAGTGTAGCCATCCTT
Mouse	Chil3 Reverse	TCTGGGTACAAGATCCCTGAA
Mouse	Arg1 Forward	TTTTTCCAGCAGACCAGCTT
Mouse	Arg1 Reverse	AGAGATTATCGGAGCGCCTT
Mouse	Mrc1 Forward	CAGGTGTGGGCTCAGGTAGT
Mouse	Mrc1 Reverse	TGGCATGTCCTGGAATGAT
Mouse	Il10 Forward	CCAGAGCCACATGCTCCTAGA
Mouse	Il10 Reverse	GGTCCTTTGTTTGAAAGAAAGTCTTC
Mouse	β-actin Forward	GCTCTTTTCCAGCCTTCCTT
Mouse	β-actin Reverse	CTTCTGCATCCTGTCAGCAA
Human	RFX1 Forward	GATCCAAGGCGGCTACAT
Human	RFX1 Reverse	CAGCCGTCTCATAGTTGTCC
Human	TLR4 Forward	TACAAAATCCCCGACAACCTCC
Human	TLR4 Reverse	GCTGCCTAAATGCCTCAGGG
Human	CCL2 Forward	TACAAAATCCCCGACAACCTCC
Human	CCL2 Reverse	GCTGCCTAAATGCCTCAGGG
Human	IL6 Forward	ATTCGGTACATCCTCGACGGC
Human	IL6 Reverse	GCCAGTGCCTCTTTGCTGCTTT
Human	TNF Forward	CTCCTTCAGACACCCTCAACCT
Human	TNF Reverse	CGACCCTAAGCCCCCAATT
Human	IL1B Forward	TGAAAGCTCTCCACCTCCAG
Human	IL1B Reverse	TGGGATCTACACTCTCCAGC
Human	IL10 Forward	GTTGCCAAGCCTTGTCTGAG
Human	IL10 Reverse	CATTCTTCACCTGCTCCACG
Human	β-actin Forward	GCACCACACCTTCTACAATGAGC
Human	β-actin Reverse	GGATAGCACAGCCTGGATAGCAAC

Table 3. Primers for BSP

Primer	Sequence
TNF（human） outer-Forward	AGAAGGAAATAGATTATAG
TNF（human） outer-Reverse	T ATACCAACAACTACCTT
TNF（human） inner - Forward	ATGGGGACGGGGTTTAGT
TNF（human） inner - Reverse	AAATCATTCAACCAACGA
IL6（human） outer- Forward	GTTGAGTAAAAGTAAGTT
IL6（human） outer-Reverse	CGATTTCTTTACTTTACT
IL6（human） inner - Forward	CGGATTATAGTGTACGGT
IL6（human） inner - Reverse	CATAACATTTCAAAACCC
IL1B（human） outer- Forward	GAGGAATTAGGAGGAGTA
IL1B（human） outer-Reverse	CCCTCCTTTACCTCTTTA
IL1B（human） inner - Forward	AGAAGTGTTATAGGTTGGA
IL1B（human） inner - Reverse	AAAATCTCACTCTATCGC
